# Influence of the HER receptor ligand system on sensitivity to cetuximab and trastuzumab in gastric cancer cell lines

**DOI:** 10.1007/s00432-016-2308-z

**Published:** 2016-12-08

**Authors:** Julia Kneissl, Anja Hartmann, Nicole Pfarr, Franziska Erlmeier, Thomas Lorber, Simone Keller, Gwen Zwingenberger, Wilko Weichert, Birgit Luber

**Affiliations:** 1 Institut für Allgemeine Pathologie und Pathologische Anatomie, Technische Universität München, Klinikum rechts der Isar, Trogerstr. 18, 81675 Munich, Germany; 2 Institute for Pathology, University Hospital Basel, Schönbeinstrasse 40, 4031 Basel, Switzerland

**Keywords:** Gastric cancer, HER receptors, EGFR, Trastuzumab, Cetuximab, Ligand

## Abstract

**Purpose:**

Gastric cancer remains a major health concern, and improvement of the therapeutic options is crucial. Treatment with targeted therapeutics such as the EGFR-targeting antibody cetuximab or the HER2-targeting antibody trastuzumab is either ineffective or moderately effective in this disease, respectively. In this study, we analysed the involvement of the HER receptor ligands amphiregulin (AREG), epidermal growth factor (EGF), heparin-binding epidermal growth factor (HB-EGF) and transforming growth factor alpha (TGFα) in the responsiveness of gastric cancer cell lines to cetuximab and trastuzumab.

**Methods:**

A panel of 11 gastric cancer cell lines was characterized for cetuximab and trastuzumab sensitivity, ligand secretion and expression and activation of the HER receptors using WST-1 cell proliferation assays, ELISAs and Western blot analyses. We further investigated the effects of an exogenous ligand application on the cetuximab and trastuzumab sensitivity.

**Results:**

We found no correlation between TGFα secretion and the sensitivity to cetuximab or trastuzumab. For AREG, we confirmed previous results indicating that this ligand is a positive predictor of cetuximab sensitivity. Exogenous HB-EGF was effective in rescuing sensitive cell lines from inhibition of cell proliferation by both, cetuximab and trastuzumab.

**Conclusions:**

Our data indicate that HB-EGF may be a useful marker for the prediction of trastuzumab sensitivity in gastric cancer.

**Electronic supplementary material:**

The online version of this article (doi:10.1007/s00432-016-2308-z) contains supplementary material, which is available to authorized users.

## Introduction

According to estimates, in 2012, 951,000 new gastric cancer cases were diagnosed worldwide and 723,000 patients died of their disease. Hence, gastric cancer was ranked as the fifth most common cancer in the world (Ferlay et al. 2015).

Although important progress in gastric cancer prevention has been achieved in recent years, therapeutic options, especially for advanced disease, are still limited. The standard treatment for unresectable or metastatic disease is still palliative chemotherapy, generally based on a platinum/fluoropyrimidine regimen (Okines et al. 2010). Following the development of targeted cancer therapeutics, the HER receptors have been favoured as putative molecular targets in gastric tumours. Due to their frequent overexpression in tumours, research efforts especially concentrated on EGFR (HER1) and HER2 [for review: (Hinoda et al. 2004)]. Finally, the approval of the monoclonal HER2-targeted antibody trastuzumab for the treatment of advanced or metastatic gastric cancer showed the potential of targeted therapies in this illness (Bang et al. 2010).

Additionally, a phase IIa trial investigating the efficacy of the HER2-targeted monoclonal antibody pertuzumab in combination with trastuzumab, capecitabine and cisplatin in patients with HER2-positive advanced gastric cancer or cancer of the gastro-oesophageal junction was the basis for an ongoing phase III study of first-line pertuzumab, trastuzumab and chemotherapy in HER2-positive metastatic gastric and gastro-oesophageal junction cancer (JACOB, NCT01774786) (Kang et al. 2014).

In contrast, the EGFR/HER2 small-molecule inhibitor lapatinib showed only limited efficacy in treating advanced gastric cancer (Hecht et al. 2016; Lorenzen et al. 2015; Satoh et al. 2014). EGFR-targeted therapeutics have been ineffective so far: the addition of the EGFR-targeted antibody cetuximab to chemotherapy failed to show any significant benefit in the phase III EXPAND trial (Lordick et al. 2013), the addition of the anti-EGFR antibody panitumumab to chemotherapy did not improve overall survival of patients in the phase III REAL3 trial (Waddell et al. 2013), and in the SWOG 0127 trial, the small-molecule inhibitor erlotinib did not improve the outcome of patients with metastatic or unresectable gastric cancer (Dragovich et al. 2006).

The rationale for these findings is unclear and has yet to be clarified. Several different resistance mechanisms against EGFR- and HER2-targeted therapies have been discovered in recent years. In colorectal cancer, activating mutations in the *KRAS* gene were shown to be associated with therapeutic failure of cetuximab-containing regimens (Karapetis et al. 2008; Lievre et al. 2006). Recently, results were published suggesting that activating *PIK3CA* mutations are associated with reduced efficacy of trastuzumab- and lapatinib-based therapies in breast cancer patients (Majewski et al. 2015). Berns and co-authors associated *PIK3CA* mutations and low PTEN expression with a reduced progression-free survival of trastuzumab-treated breast cancer patients (Berns et al. 2007). Besides, several other resistance mechanisms against HER2-targeted therapeutics have been proposed, including enhanced expression and activation of HER3 and functional crosstalk with the receptor tyrosine kinase MET [for review: (Shimoyama 2014)]. In addition to other receptor tyrosine kinases and the downstream signalling pathways, the ligand system of the HER receptors has been spotlighted as a potential source for resistance mechanisms against HER receptor-targeting therapeutics. Among the family of HER receptor ligands, amphiregulin (AREG) and epiregulin in particular have been studied for their involvement in the responsiveness of tumours to cetuximab-containing regimens (Baker et al. 2011; Cushman et al. 2015; Jacobs et al. 2009; Jonker et al. 2014; Khambata-Ford et al. 2007; Pentheroudakis et al. 2013; Takahashi et al. 2014; Yoshida et al. 2013). Although HER2 does not possess a functional ligand-binding domain, some findings suggest that the HER receptor ligand system is involved in trastuzumab resistance as well (Kim et al. 2015; Ritter et al. 2007; Valabrega et al. 2005; Yotsumoto et al. 2010). These studies focused mainly on cetuximab treatment of colorectal cancer and tumours of the head and neck as well as trastuzumab treatment in breast cancer. To expand these data, the aim of our study was to investigate the role of the HER receptor ligand system in the responsiveness of gastric cancer cells to cetuximab and trastuzumab, with special focus on AREG, transforming growth factor alpha (TGFα) and heparin-binding epidermal growth factor (HB-EGF).

## Materials and methods

### Cell lines and cell culture conditions

The cell lines AGS, Hs746T, KATOIII, LMSU, MKN1, MKN28 and MKN45 were obtained and cultured as described previously (Heindl et al. 2012; Kneissl et al. 2012). The cell lines GSU, H111TC, HGC-27 and MKN7 were provided by the Cell Bank RIKEN BioResource Center (Tsukuba, Japan), and the identity of the cell lines was guaranteed by the provider. GSU, H111TC and MKN7 cells were grown in RPMI-1640 medium (Invitrogen/Gibco, Darmstadt, Germany), and HGC-27 cells were cultured in Eagle’s minimum essential medium (MEM, Sigma-Aldrich Chemie GmbH, Taufkirchen, Germany). Both media were supplemented with 10% foetal bovine serum Sera Plus (PAN Biotech, Aidenbach, Germany) and penicillin–streptomycin (PAA Laboratories, Pasching, Austria; 100 international units (IU)/ml, 100 μg/ml); in addition, RPMI-1640 was supplemented with 2 mM l-glutamine (Invitrogen/Gibco). General cultivation conditions and routine mycoplasma testing as well as cell line validation were performed as described previously (Heindl et al. 2012; Kneissl et al. 2012).

### Antibodies and reagents

For Western blot analysis, the following antibodies were used: anti-EGFR (Cell Signaling, Leiden, NL, #2232), anti-pEGFR (Y1068) (Invitrogen, #44788G), anti-HER2 (Cell Signaling, #2165), anti-pHER2 (Y1248) (Cell Signaling, #2247), anti-HER3 (Cell Signaling, #4754), anti-pHER3 (Y1222) (Cell Signaling, #4784), anti-HER4 (Cell Signaling, #4795), anti-pHER4 (Y1284) (Cell Signaling, #4757), anti-TACE (Cell Signaling, #6978), anti-β-actin (Sigma-Aldrich, #A1978), anti-α-tubulin (Sigma-Aldrich, #T9026), anti-rabbit IgG (Cell Signaling, #7074) and anti-mouse IgG (GE Healthcare, Munich, Germany, #NA931).

The following monoclonal therapeutic antibodies were used: cetuximab (Erbitux™, Merck Serono, Darmstadt, Germany), trastuzumab (Herceptin™, Roche, Penzberg, Germany) and isotype control (Southern Biotech, Birmingham, USA, #0151K-14). The corresponding solvent controls were as follows: solvent control isotype: 1 × PBS; solvent control trastuzumab: 3.36 mg l-histidine HCl, 2.16 mg l-histidine, 136.2 mg trehalose dihydrate, 0.6 mg polysorbate 20, dissolved in 7.2 ml sterile water (http://www.ema.europa.eu/docs/en_GB/document_library/EPAR_-_Scientific_Discussion/human/000278/WC500049816.pdf).

The solvent control for cetuximab was described previously (Heindl et al. 2012).

Recombinant ligands were obtained as follows: human AREG (R&D systems, Minneapolis, USA, #262-AR-100), human HB-EGF (Pelobiotech, Planegg, Germany; #PB-Z3051) and human EGF (Sigma-Aldrich, #E9644).

Chemotherapeutics were obtained as follows: 5-fluorouracil (Sigma-Aldrich, #F6627), cisplatin (Sigma-Aldrich, #P4394).

### WST-1 cell proliferation assay

To assess the effects of the treatments on cell proliferation, the WST-1 cell proliferation assay was used according to the manufacturer’s instructions (Roche Diagnostics, Mannheim, Germany; #11644807001). All samples were analysed in triplicate. Cells were seeded at densities between 0.5 × 10^3^ and 5 × 10^3^ cells per well in 80 µl culture medium and allowed to settle for 24 h. The following day, cetuximab and/or trastuzumab and/or recombinant human AREG, EGF or HB-EGF and/or chemotherapeutics were added to a final volume of 100 µl per well. The isotype control was added to a final concentration of 100 µg/ml for cetuximab and 40 µg/ml for trastuzumab. Due to the high volume needed, we investigated the effect of the isotype solvent as well. The applied volume for the cetuximab solvent corresponded to 100 µg/ml cetuximab, and the trastuzumab solvent corresponded to 40 µg/ml trastuzumab. Assays using cetuximab were incubated for 48 h, and assays with trastuzumab were incubated for 72 h. Experiments with concomitant treatment of cetuximab and trastuzumab were incubated for 72 h as well. After this incubation period, pre-warmed WST-1 reagent was added. The absorbance of the samples was measured after an incubation period between 30 min and 2 h, depending on the cell line. An Asys Expert Plus microplate reader was used for measurements (Biochrom, Berlin, Germany).

### Extraction of genomic DNA

For extraction of genomic DNA from GSU and H111TC cells, the DNeasy kit was used according to manufacturer’s instructions (Qiagen, Hilden, Germany; #69504). The DNA concentration was measured using the QuBit 2.0 DNA high sensitivity kit (Thermo Fisher Scientific, Waltham, USA, #Q32854). Furthermore, DNA sequencing grade quality was determined by a qPCR assay (RNAse P assay, Thermo Fisher Scientific, Waltham, USA, #4316831) as described previously (Endris et al. 2013).

### Library preparation and semiconductor sequencing

For library preparation, the multiplex PCR-based Ion Torrent AmpliSeq™ technology (Thermo Fisher Scientific, Waltham, USA), together with the Cancer Hotspot Panel (CHPv2; Thermo Fisher Scientific, #4475346), was used as described previously (Endris et al. 2013; Stenzinger et al. 2014).

Amplicon library preparation was performed with the Ion AmpliSeq Library Kit v2.0 (Thermo Fisher Scientific, #4480442). For mutation analysis, the CHPv2 panel, which consists of one primer pool yielding 207 amplicons covering hot spot regions of 50 known cancer-related genes, was employed. For amplification, approximately 10 ng of DNA, as determined by qPCR assay, was used. Briefly, the DNA was mixed with the primer pool and the AmpliSeq HiFi Master Mix in a 20-µl reaction volume and transferred to a PCR cycler (Biometra, Göttingen, Germany). After the end of the PCR, amplicons were partially digested using FuPa reagent, followed by the ligation of barcoded sequencing adapters (Ion Xpress Barcode Adapters, Thermo Fisher Scientific, #4474517). The final library was purified using AMPure XP magnetic beads (Beckman Coulter, Krefeld, Germany, # A63880) and quantified using qPCR (Ion Library Quantitation Kit, Thermo Fisher Scientific, #4468802) on a StepOne*Plus* qPCR machine (Thermo Fisher Scientific, Waltham, USA). The individual libraries were diluted to a final concentration of 100 pM. All libraries were pooled and processed for library amplification on Ion Spheres using Ion PGM™ Template OT2 200 Kit **(**Thermo Fisher Scientific, #4480974). Unenriched libraries were quality controlled using an Ion Sphere quality control measurement on a QuBit instrument. After library enrichment (Ion OneTouch ES, Thermo Fisher Scientific), the library was processed for sequencing using the Ion PGM™ Sequencing 200 Kit v2 chemistry (Thermo Fisher Scientific, #4482006) and the barcoded libraries were loaded onto a 318v2 chip.

### Data analysis

Raw sequencing data were processed using the implemented Torrent Suite software (version 4.4.3) and aligned with the human genome (version hg19) using the TMAP algorithm. For DNA mutation analysis, the aligned reads were processed using the built-in Variant Caller plugin (version 4.4.3). Variant annotation was performed using a custom-build variant annotation pipeline in the CLC Genomics Workbench (version 8.0.2). For visualization of sequencing and fusion reads, the Integrative Genomic Browser (IGV, http://www.broadinstitute.org/igv/) was used. We used the COSMIC (catalogue of somatic mutations in cancer) database (Forbes et al. 2015; Sherry et al. 2001) to identify already known somatic mutations and mutation types, respectively.

### Prediction of copy number alterations

Copy number variations (CNVs; amplifications and deletions) were identified using the coverage data summary for each sample and each amplicon generated by the Torrent Suite software. Detection of CNVs was performed according to Endris et al. (2013). In brief, gene amplifications and/or deletions were determined by a simple algorithm using the number of reads of each individual amplicon in the sequenced sample: (i) the number of reads of each individual pool amplicon was divided by the total number of sequencing reads of the respective sample = (reads amplicon x/total reads) = normalized amplicon read depth value (NARD); (ii) the NARD was multiplied by the total number of amplicons (e.g. lung cancer panel = 140 amplicons; NARD (reads amplicon x/total reads) × 140); (iii) the median normalized amplicon read depth (MNARD) was determined for all samples = median (NARDSample1:NARDSampleX), reflecting the typical amplification efficiency of each individual amplicon in the pool; and iv) the standard deviation (SD) was determined from the median value. Amplifications are considered true if the NARDs of all amplicons covering a gene differ by >2 SD from the median value. However, deletions are considered true if the SD of all amplicons covering a gene is <0.5.

### Array-comparative genomic hybridization

Array-comparative genomic hybridization (aCGH) was performed as previously described (Juskevicius et al. 2016; Ruiz et al. 2011), with minor modifications. In brief, 500 ng of sample DNA (cell line DNA of GSU or H111TC) and equal amounts of female reference genomic DNA (Promega, Madison, WI, USA) were digested with DNaseI to a size range of 200–500 bp. Subsequent labelling of sample and reference DNA with Cy3-dUTP and Cy5-dUTP, respectively, was performed with the BioPrime^®^ Array CGH Genomic Labeling System (Invitrogen, Carlsbad, CA, USA). The success of labelling was assessed by quantifying the specific activities of the incorporated dyes with a Nanodrop (Thermo Fischer Scientific, Waltham, MA, USA). Reference and sample DNA were mixed and hybridized to 180 k CGH arrays (Agilent Technologies, Santa Clara, CA, USA) for 24 h in a rotating oven at 67 °C. Microarray slides were scanned with the Agilent 2565C DNA scanner, and images were analysed with Agilent’s Feature Extraction using default settings. Feature extracted array CGH data were evaluated using Agilent’s CytoGenomics software v3.0.1.1. Aberrations were called with the aberration detection algorithm ADM2 set to a threshold of 12.0, with fuzzy zero and GC-content (window size: 2 kb) correction. A minimum of three probes were necessary to call an aberration.

### ELISA

For detection of human AREG, HB-EGF and TGFα, DuoSet ELISAs were used (R&D systems, Minneapolis, USA; #DY262, DY259, DY239) according to the manufacturer’s instruction. All samples were analysed in duplicates.

To determine the levels of secreted ligand (AREG, HB-EGF, TGFα), 1 × 10^6^ cells were seeded into 10 ml medium and incubated for 24 h. Next, conditioned cell culture medium was harvested and centrifuged at maximum speed (4 °C) for 10 min to remove cell debris. Aliquots were stored at −80 °C.

For analysis of the effect of trastuzumab treatment in combination with an exogenous ligand application on ligand secretion, 2 × 10^5^ cells (MKN45, GSU) were seeded into 2 ml medium. Cells were allowed to settle overnight. The following day, the cells were treated with medium containing 10 µg/ml trastuzumab and/or 15 ng/ml AREG or 0.1 ng/ml EGF or 0.4 ng/ml HB-EGF for 6 h. The conditioned medium was harvested and stored as mentioned above.

The effect of extensive trastuzumab and cetuximab treatment was analysed by seeding 2 × 10^5^ cells into 6 ml medium. After 24 h, 10 µg/ml cetuximab or trastuzumab was added. The cells were incubated for 8 days with medium changes every 3–4 days. Then, the cells were trypsinized and 2 × 10^5^ cells were seeded into 2 ml medium containing 10 µg/ml cetuximab or trastuzumab. Conditioned medium was harvested after 24 h as mentioned above.

### Western blot

For analysis of the effect of trastuzumab treatment in combination with an exogenous ligand on protein levels, 2 × 10^5^ cells (MKN45, GSU) were seeded into 2 ml medium. Cells were allowed to settle overnight. The following day, the cells were treated with medium containing 10 µg/ml trastuzumab and/or 15 ng/ml AREG, 0.1 ng/ml EGF or 0.4 ng/ml HB-EGF for 6 h. The effect of extensive trastuzumab and cetuximab treatment was analysed by seeding 2 × 10^5^ cells into 6 ml medium. After 24 h, 10 µg/ml cetuximab or trastuzumab was added. The cells were incubated for 8 days with medium changes every 3–4 days. Cell lysates were prepared as described previously (Bremm et al. 2008). Western blot analysis was performed using a standard protocol described previously. The antibodies were used in the following dilutions: anti-EGFR, anti-HER2, anti-pHER2, anti-HER3, anti-pHER3, anti-HER4 and anti-pHER4: 1:1000 in 5% BSA-TBS-T (w/v); anti-pEGFR: 1:2000 in 5% milk-TBS-T (w/v); anti-TACE: 1:3000 in 5% milk-TBS-T (w/v); anti-β-actin, anti-α-tubulin, anti-mouse IgG: 1:10,000 in 5% milk-TBS-T (w/v); anti-rabbit IgG: 1:2000 in TBS-T. Signal detection was performed using an enhanced chemiluminescence reaction. The signals were quantified by densitometric measurement via ImageJ software 1.42q (National Institute of Health, MD, USA).

### Literature search

A PubMed search was performed for relevant gastric cancer-based literature about the HER receptor ligands AREG, EGF, HB-EGF and TGFα (date: 6 May 2015). The search was performed using the following terms: amphiregulin gastric cancer, EGF gastric cancer, HB-EGF gastric cancer, TGFα gastric cancer.

Inclusion criteria were as follows: (1) published as an original article, (2) published in the English language, (3) full-text access, (4) examined ligand mRNA levels, ligand protein levels or pro-ligand protein levels in (5) tissue, serum, gastric juice and other body fluids of gastric cancer patients. Studies dealing only with tumours of the gastroesophageal junction (GEJ) were excluded as well as studies based on less than 10 patients and studies with an unclear number of tumour samples. Furthermore, studies concerning gene polymorphisms in ligand genes and their association with gastric cancer risk were excluded. Additionally, all studies regarding only co-expression of HER ligands in combination with other proteins were not included. The studies were screened for relevant information regarding the expression rates of the ligands and their correlation to clinicopathologic features.

### Statistical analysis

All analyses presented herein were performed in at least three independent experiments. Statistical analyses were calculated using IBM SPSS Statistics 22 (IBM, Armonk, NY, USA). The results are shown as the mean value ± standard deviation (SD). For comparing pairs of different treatment conditions, the two-sided Welch *t* test was used. When values were compared to a reference value (=100%), we used the one-sample *t* test. *p* values ≤0.05 are indicated by * and ≤0.01 by **. The authors will provide all statistical analyses on request.

## Results

### Trastuzumab and cetuximab sensitivity of gastric cancer cell lines

In this study, a panel of 11 gastric cancer cell lines was used. In addition to the data we published recently (Heindl et al. 2012; Kneissl et al. 2012), we identified the cell lines GSU, H111TC and MKN7 as cetuximab sensitive (Table 1, Online Resource 1). Following treatment with 10 µg/ml cetuximab, GSU cells displayed a decrease in cell proliferation to 68.10% in comparison with the untreated control (*p* = 0.04). H111TC cells were less sensitive with growth rates of 82.30% after treatment with 10 µg/ml cetuximab (*p* = 0.002) and a maximum inhibition to 80.16% after application of 200 µg/ml (*p* < 0.001). MKN7 cells showed only a minor sensitivity with a maximum decrease of cell proliferation to 83.63% following treatment with 100 µg/ml cetuximab (*p* = 0.001). HGC-27 cells were completely resistant to cetuximab.

**Table 1:** Molecular and physiological characteristics of gastric cancer cell lines used in this study

Cell line	Cetuximab sensitivity	AREG secretion	*KRAS* mutation	*PIK3CA* mutation	*HER2* amplification
AGS	− (Heindl et al. 2012)	− (Kneissl et al. 2012)	G12D (Kim et al. 2003)	E545A^7^ (Mita et al. 2009)	Not amplified (Wainberg et al. 2010)
GSU	**+++** ^1^	**+++** ^2^	**G12D** ^3^	**WT** ^7^	**No suspected copy number variations** ^9^ **Not amplified** ^**10**^
H111TC	**++** ^1^	**+++** ^2^	**WT** ^3^	**WT** ^7^	**Suspected copy number variations** ^9^ **Amplification** ^**10**^
HGC-27	**−** ^1^	**−** ^2^	WT^5^ (Kubo et al. 2009)	E542K^7^ (Zhou et al. 2011)	Not amplified (Nam et al. 2012)
Hs746T	−	+ (Kneissl et al. 2012)	WT^6^ (Kneissl et al. 2012)	WT^7^ (Kneissl et al. 2012)	Not amplified (Zang et al. 2011)
KATOIII	− (Heindl et al. 2012)	++	WT^5^ (Kubo et al. 2009)	WT^8^ (Li et al. 2013)	Not amplified (Wainberg et al. 2010)
LMSU	−	− (Kneissl et al. 2012)	WT^6^ (Kneissl et al. 2012)	WT^7^ (Kneissl et al. 2012)	ND
MKN1	++	++ (Kneissl et al. 2012)	Amp (Mita et al. 2009)	E545K^7^ (Mita et al. 2009)	Not amplified (Wainberg et al. 2010)
MKN7	**+** ^1^	**++** ^2^	WT^4^ (Mita et al. 2009)	ND	Amplification (Fukushige et al. 1986)
MKN28	++ (Heindl et al. 2012)	++ (Kneissl et al. 2012)	WT^4^ (Mita et al. 2009)	ND	Not amplified (Zang et al. 2011) No high-level amplification (Takada et al. 2005)
MKN45	− (Heindl et al. 2012)	− (Kneissl et al. 2012)	WT^5,4^ (Kubo et al. 2009; Mita et al. 2009)	H1047R^7^ (Zhou et al. 2011)	Not amplified (Nam et al. 2012)

Bold = results obtained in this study; ^1^ online resource 1; ^2^ online resource 7; ^3^ exon 2–4; ^4^ codon 12/13; ^5^ exon 1/2; ^6^ exon 2; ^7^ exons 9, 20; ^8^ screening for E542K, E545D, E545K, H1047R, H1047L; ^9^ online resource 2; ^10^ online resource 3, 4; *ND* not described

**Fig. 1:**
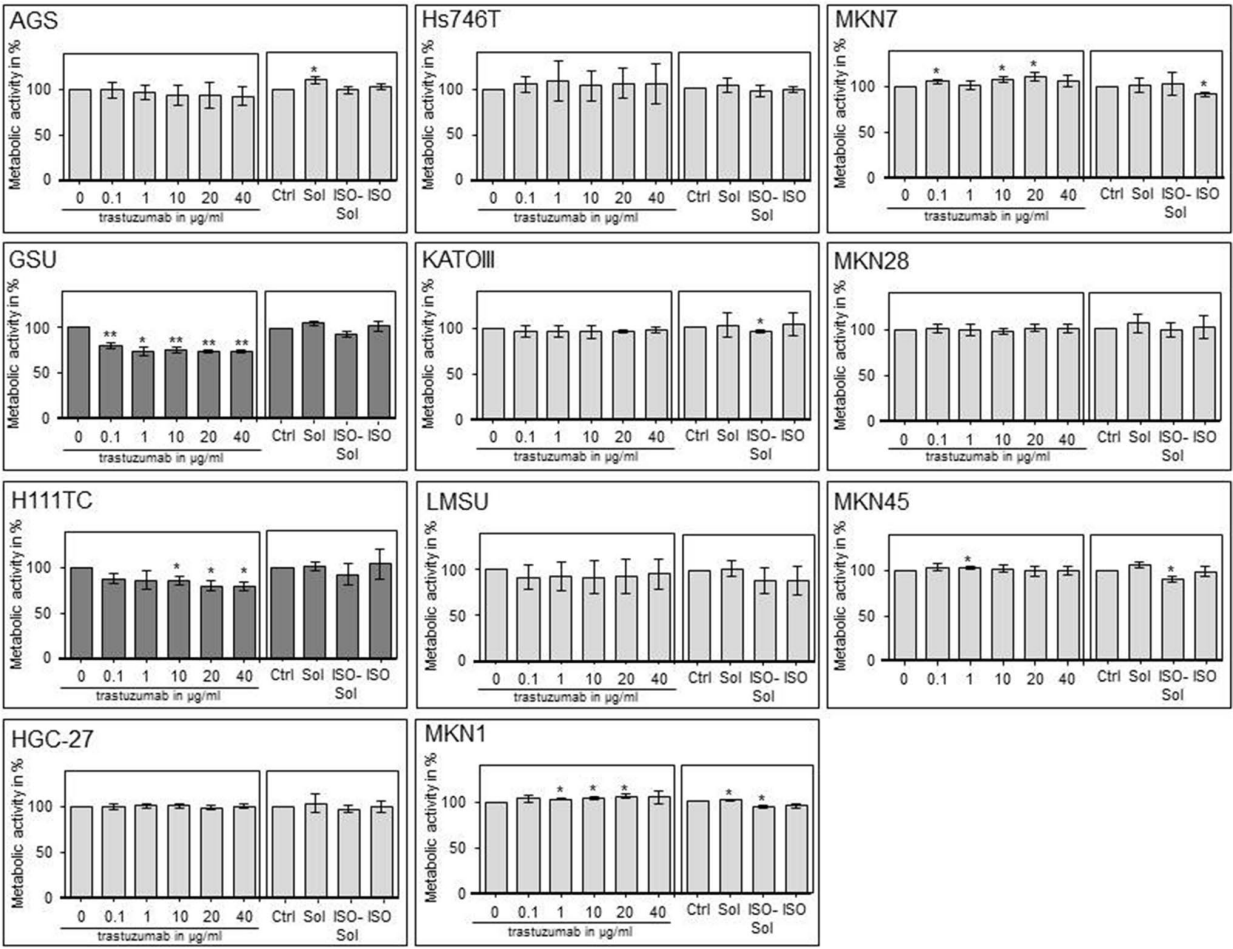
Effect of trastuzumab treatment on the cell proliferation of gastric cancer cell lines. The gastric cell lines AGS, GSU, H111TC, HGC-27, Hs746T, KATOIII, LMSU, MKN1, MKN7, MKN28 and MKN45 were treated for 72 h with the indicated amounts of trastuzumab (0/0.1/1/10/20/40 µg/ml), a solvent control (Sol), an isotype control (ISO) or isotype solvent control (ISO-Sol). Afterwards, the metabolic activity of the cell lines was determined via WST-1 cell proliferation assay. Only GSU and H111TC cells were trastuzumab sensitive (*dark grey diagrams*). The mean value of at least three independent experiments is shown. *p* values at significance levels of ≤0.050 and ≤0.010 are indicated by (*) and (**), respectively

Additionally, we investigated the trastuzumab sensitivity of the cell lines via cell proliferation assay (Fig. 1). We identified two cell lines as trastuzumab sensitive: GSU and H111TC. Both displayed a significant decrease in cell proliferation after application of the therapeutic. For GSU, 0.1 µg/ml was already sufficient to inhibit the proliferation rate to 80.10% (*p* = 0.009), while 40 µg/ml caused an inhibition to 72.98% (*p* = 0.002). H111TC displayed a less sensitive phenotype with a maximum inhibition to 80.03 and 80.31% after application of 20 µg/ml and 40 µg/ml trastuzumab (*p* = 0.024; *p* = 0.02), respectively. For the other cell lines, no significant inhibition of cell proliferation was observed.

### Genetic alterations in gastric cancer cell lines

Among the numerous genetic alterations which have been connected to resistance to cetuximab, activating mutations in the *KRAS* gene are especially important in colorectal cancer regimens (Karapetis et al. 2008; Lievre et al. 2006). Regarding trastuzumab therapy, activating mutations in the *PIK3CA* gene have been described to be associated with a poorer therapy outcome in breast cancer patients (Berns et al. 2007; Majewski et al. 2015). Additionally, the existence of *HER2* amplifications and the resulting consecutive overexpression of the protein are crucial factors in the response predictions for trastuzumab-based regimens in patients with gastric cancer (Okines et al. 2010). Therefore, we collected information about these three genetic loci in our panel of gastric cancer cell lines. We were able to extract some data from the literature and completed the missing data for the majority of the cell lines with our own analyses (Table 1).

Interestingly, we found that the cetuximab-sensitive cell line GSU harbours an activating *KRAS* mutation in exon 2 (G12D), and MKN1 cells, which are cetuximab sensitive, were described as *KRAS* amplified (Mita et al. 2009). However, activating *PIK3CA* mutations have only been described for trastuzumab-insensitive cell lines (MKN1, HGC-27, AGS, MKN45) (Mita et al. 2009; Zhou et al. 2011), while we found both sensitive cell lines, GSU and H111TC, to be wild type. Regarding the *HER2*-amplification status, the picture was less consistent as we found a suspected *HER2* amplification only for the cell line H111TC by next-generation sequencing (Online Resource 2) which was confirmed by array-comparative genomic hybridization (*ERBB2*/CEP7 ratio > 2.0) (Online Resource 3, 4).

### Enhanced inhibition of gastric cancer cell growth by trastuzumab in combination with chemotherapeutics but not with cetuximab

In our cells, trastuzumab monotreatment showed only a moderate effect on cell proliferation. Therefore, we decided to investigate the effect of a concomitant application of cetuximab or chemotherapeutics (5-fluorouracil [5-FU] and cisplatin) to trastuzumab.

**Fig. 2:**
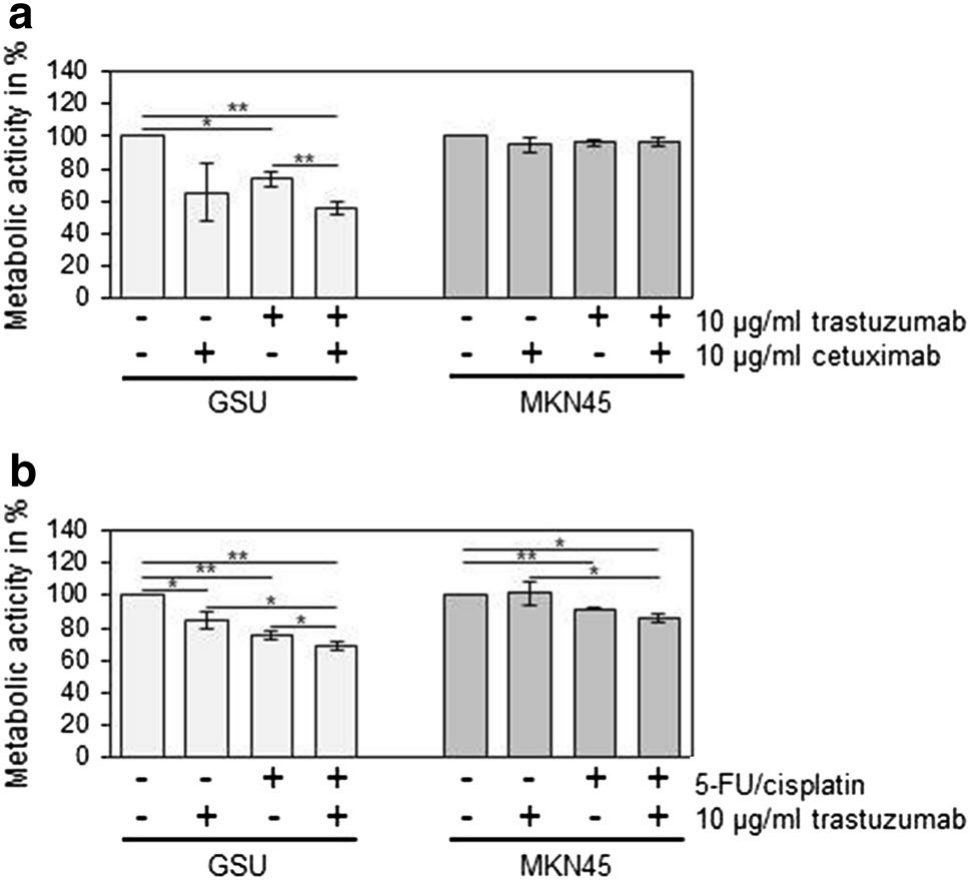
Effect of concomitant treatment of gastric cancer cells with trastuzumab and chemotherapeutics or cetuximab on cell proliferation. GSU and MKN45 cells were treated for 72 h with 10 µg/ml trastuzumab and/or 10 µg/ml cetuximab (**a**) or chemotherapeutics (25 ng/ml cisplatin, 2.5 ng/ml 5-FU;** b**). The metabolic activity of the cells was measured using the WST-1 cell proliferation assay. For concomitant trastuzumab and cetuximab treatment, no increased inhibitory effect in comparison with the monotreatment was observed. However, addition of chemotherapeutics to trastuzumab treatment yielded in GSU cells in an enhanced inhibition of cell proliferation, compared to trastuzumab or chemotherapeutics alone. The mean value of three independent experiments is shown. *p* values at significance levels of ≤0.050 and ≤0.010 are indicated by (*) and (**), respectively

Although GSU cells are sensitive to trastuzumab as well as to cetuximab, a combination of both monoclonal antibodies failed to show any enhancing effect in comparison with the monotreatment (Fig. 2a). However, the addition of 5-FU and cisplatin to trastuzumab led to significantly stronger growth inhibition in GSU cells than trastuzumab or chemotherapy alone (Fig. 2b). This effect was not observed in the trastuzumab-insensitive cell line MKN45, although this cell line reacted to the monotreatment with chemotherapeutics with a significant growth inhibition (*p* = 0.02).

### Effects of extended trastuzumab and cetuximab treatment on the expression and activation of HER receptors

**Fig. 3:**
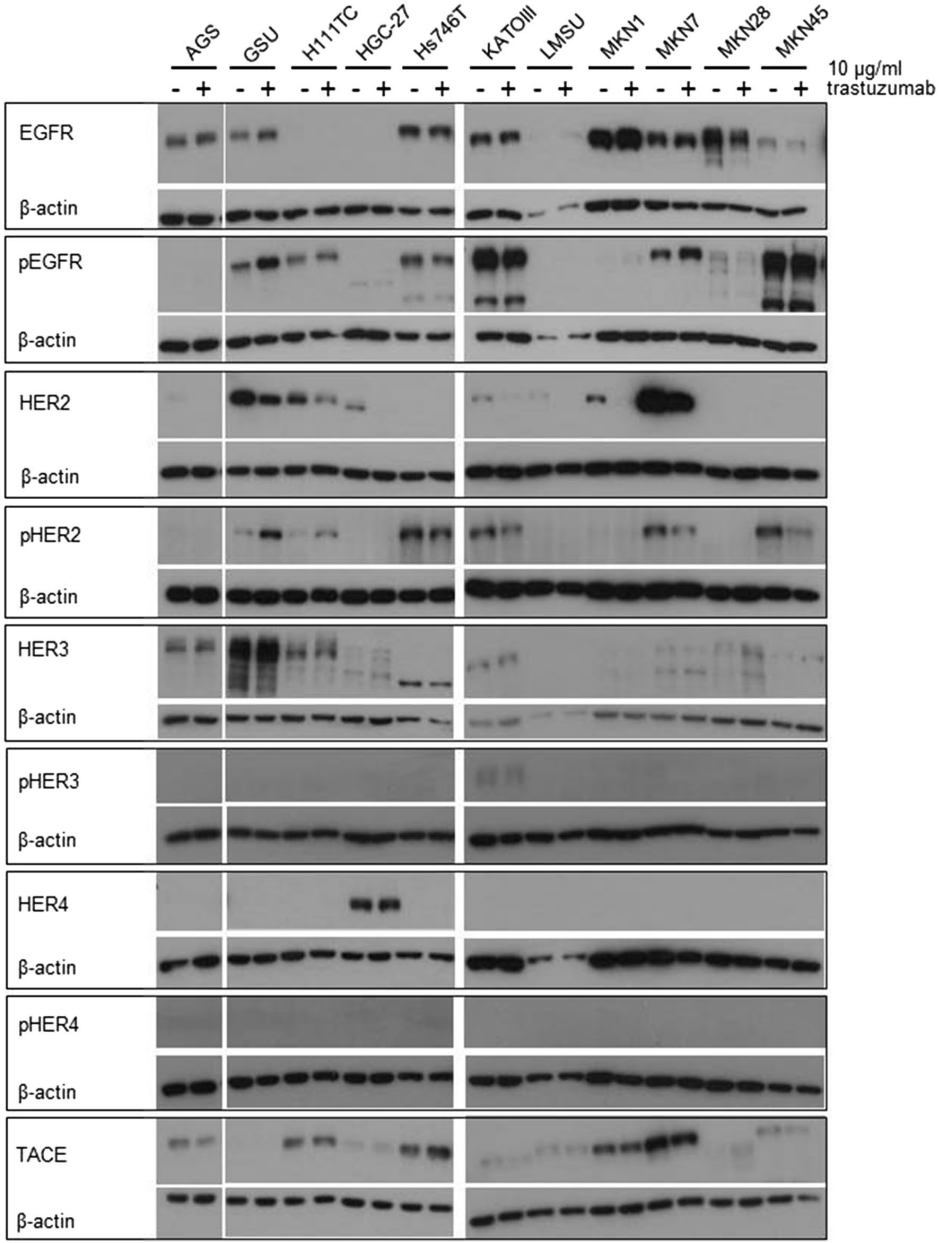
Effect of treatment with trastuzumab for 8 days on the expression profile of HER and pHER receptors. All gastric cancer cell lines were treated for 8 days with 10 µg/ml trastuzumab; afterwards, the expression of EGFR, HER2, HER3, HER4, pEGFR, pHER2, pHER3 and pHER4 was determined via Western blot analysis. Basal expression of HER receptors varied highly between the cell lines. No obvious correlation between basal HER/pHER receptor expression and/or changes in expression and responsiveness to trastuzumab could be observed

**Fig. 4:**
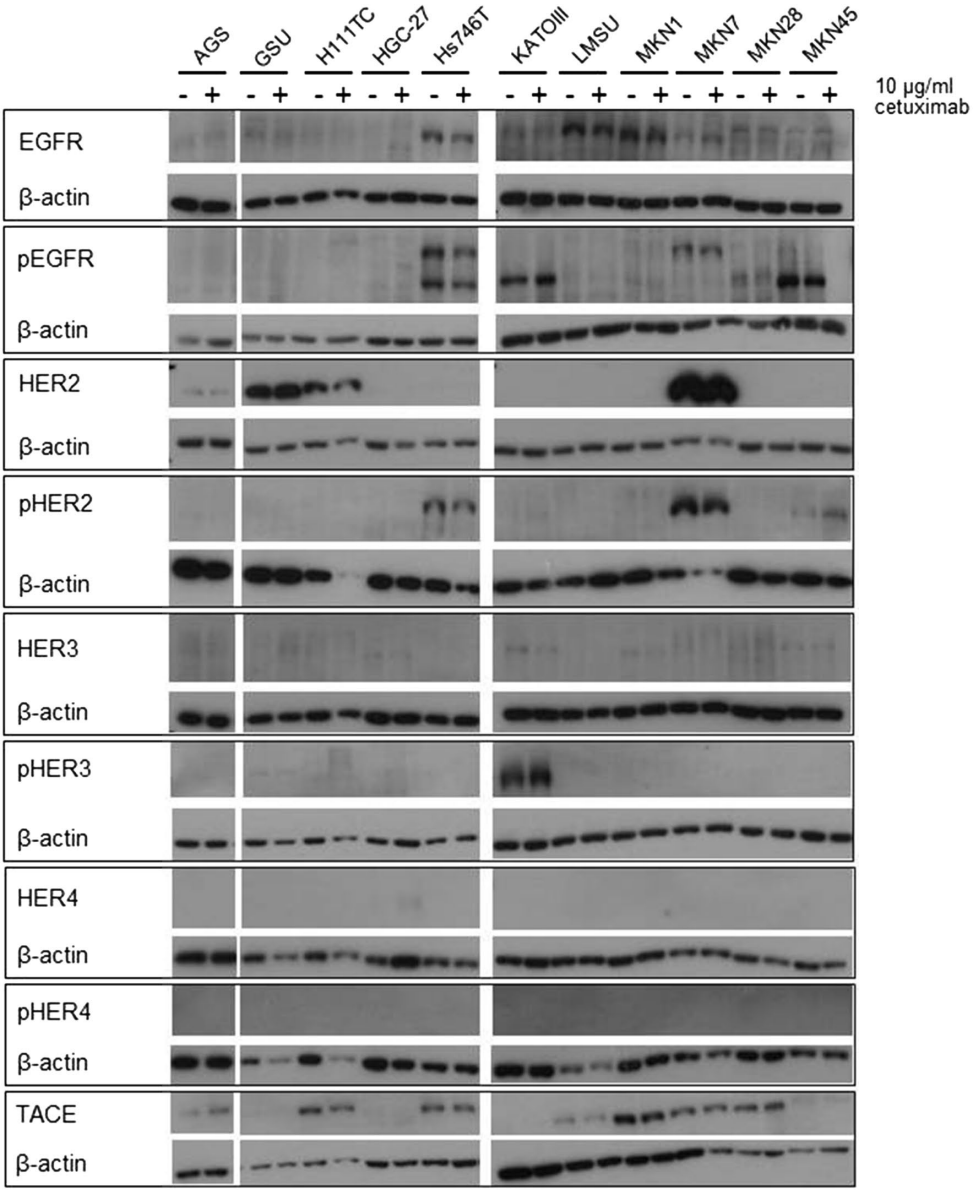
Effect of treatment with cetuximab for 8 days on the expression profile of HER and pHER receptors. All gastric cancer cell lines were treated for 8 days with 10 µg/ml cetuximab; afterwards, the expression of EGFR, HER2, HER3, HER4, pEGFR, pHER2, pHER3 and pHER4 was determined via Western blot analysis. Only minor effects of the treatment on HER/pHER receptor profile were detected. No obvious correlation between basal HER/pHER receptor expression and/or changes in expression and responsiveness to cetuximab could be observed

To determine the effect of an extended application of trastuzumab and cetuximab on the expression and activation of the HER receptors, we treated all cell lines with the therapeutics for 8 days and measured the expression of EGFR, pEGFR, HER2, pHER2, HER3, pHER3, HER4 and pHER4 (Figs. 3, 4, Online Resource 5, 6). The basal expression rate of EGFR, pEGFR, HER2, pHER2, HER3 and pHER3 varied highly between the cell lines. The highest EGFR levels were expressed by MKN1 cells; AGS, GSU, Hs746T, KATOIII, MKN7 and MKN28 cells displayed medium expression levels, and in MKN45, LMSU, H111TC and HGC-27 cells, EGFR was hardly detectable. In contrast to these findings, pEGFR was expressed at the highest levels by KATOIII and MKN45 cells, a medium expression rate was detected in GSU, H111TC, Hs746T and MKN7 cells, and pEGFR was barely detectable in AGS, HGC-27, LMSU, MKN1 and MKN28 cells. For AGS, Hs746T, KATOIII, LMSU, MKN1, MKN28 and MKN45, these findings support our recently published results (Heindl et al. 2012; Kneissl et al. 2012). Regarding HER2, the highest levels were expressed by MKN7 cells, medium levels were expressed by GSU, H111TC, HGC-27, KATOIII, LMSU and MKN1 cells, and HER2 expression was hardly detectable in AGS, Hs746T, MKN28 and MKN45 cells. We were able to detect medium levels of pHER2 in GSU, H111TC, Hs746T KATOIII, MKN7 and MKN45 cells, while in all other cell lines, the protein was hardly detectable. All cell lines, with the exception of LMSU (no expression) and GSU (high expression), expressed HER3 at median levels; however, we were only able to regularly detect pHER3 above the background level in KATOIII cells. pHER4 could not be detected in any cell line, and only HGC-27 cells displayed a medium expression of HER4. Regarding the effect of 8 days of cetuximab treatment on the expression levels, we found no obvious patterns for EGFR, pEGFR, HER3 and pHER3. However, densitometric measurement revealed an increase in the expression of HER2 after treatment for 8 of 11 cell lines (significant for H111TC and MKN7 cells; *p* = 0.041; *p* = 0.029). pHER2 expression was enhanced in 10 out of 11 cell lines, but this effect was only significant for MKN7 (*p* = 0.028). Regarding the effect of 8 days of trastuzumab treatment, relevant patterns were only observed for HER2. H111TC, HGC-27, MKN1 and MKN28 all displayed a significant decrease in HER2 expression after trastuzumab treatment (*p* = 0.003; 0.01; 0.038; 0.015, respectively). Furthermore, pHER3 levels significantly decreased in Hs746T and MKN7 cells. Additionally, we investigated the effect of the trastuzumab and cetuximab treatment on the expression of the protein TACE which is responsible for the cleavage of the membrane-bound ligand precursors into the soluble ligand [for review: (Mochizuki and Okada 2007; Sternlicht and Sunnarborg 2008)]. We detected only for GSU cells after trastuzumab treatment a significant decrease indicating only a minor influence of cetuximab or trastuzumab treatment on TACE expression in gastric cancer cell lines.

### HER receptor ligand level in gastric cancer cell lines

We analysed the level of HB-EGF and TGFα in our panel of gastric cancer cell lines. For most cell lines, AREG secretion had already been determined in a previous study (Table 1; Kneissl et al. 2012). In addition, we found that GSU, H111TC and MKN7 cells secrete high amounts of AREG to the medium, while the AREG secretion of HGC-27 was barely detectable (Table 1, Online Resource 7).

**Fig. 5:**
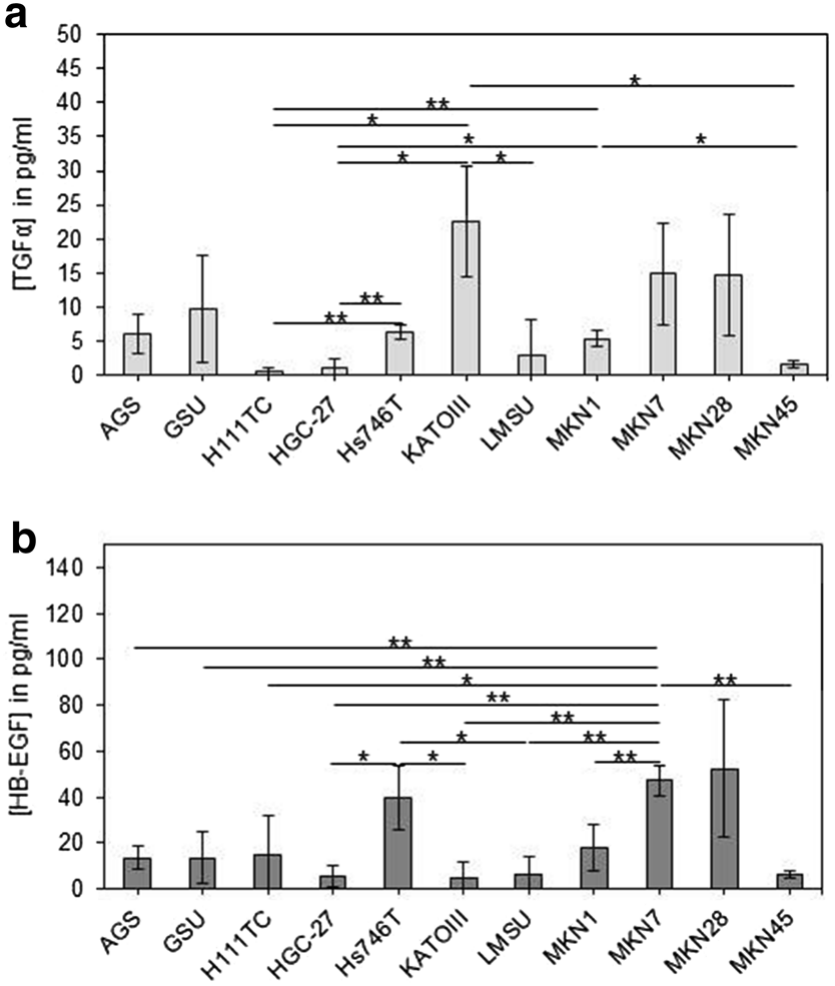
Secretion of TGFα and HB-EGF by gastric cancer cell lines. Cells were incubated for 24 h before the amount of secreted TGFα (**a**) or HB-EGF (**b**) was measured in the conditioned medium by ELISA. The highest concentrations of TGFα were detected for KATOIII, MKN7 and MKN28 cells. For all other cell lines, TGFα levels were below 10 pg/ml. The highest concentrations of HB-EGF were detected for Hs746T, MKN7 and MKN28 cells. For all other cell lines, HB-EGF levels were below 20 pg/ml. The mean value of three independent experiments is shown. *p* values at significance levels of ≤0.050 and ≤0.010 are indicated by (*) and (**), respectively

TGFα levels in the conditioned medium of the cell cultures were analysed, and a broad range was detected (Fig. 5a). TGFα was hardly detectable with values below 5 pg/ml in H111TC, HGC-27, LMSU and MKN45 cells. The cell lines AGS, GSU, Hs746T and MKN1 secreted medium levels of TGFα in a range between 5 and 10 pg/ml in the medium. The highest concentrations were detected for KATOIII, MKN7 and MKN28 cells.

HB-EGF was secreted in different concentrations; however, for the majority of the cell lines, HB-EGF levels were below 20 pg/ml. Substantially higher concentrations were only secreted by Hs746T, MKN7 and MKN28 cells (Fig. 5b).

### Effects of prolonged trastuzumab and cetuximab treatment on HER receptor ligand secretion

**Fig. 6:**
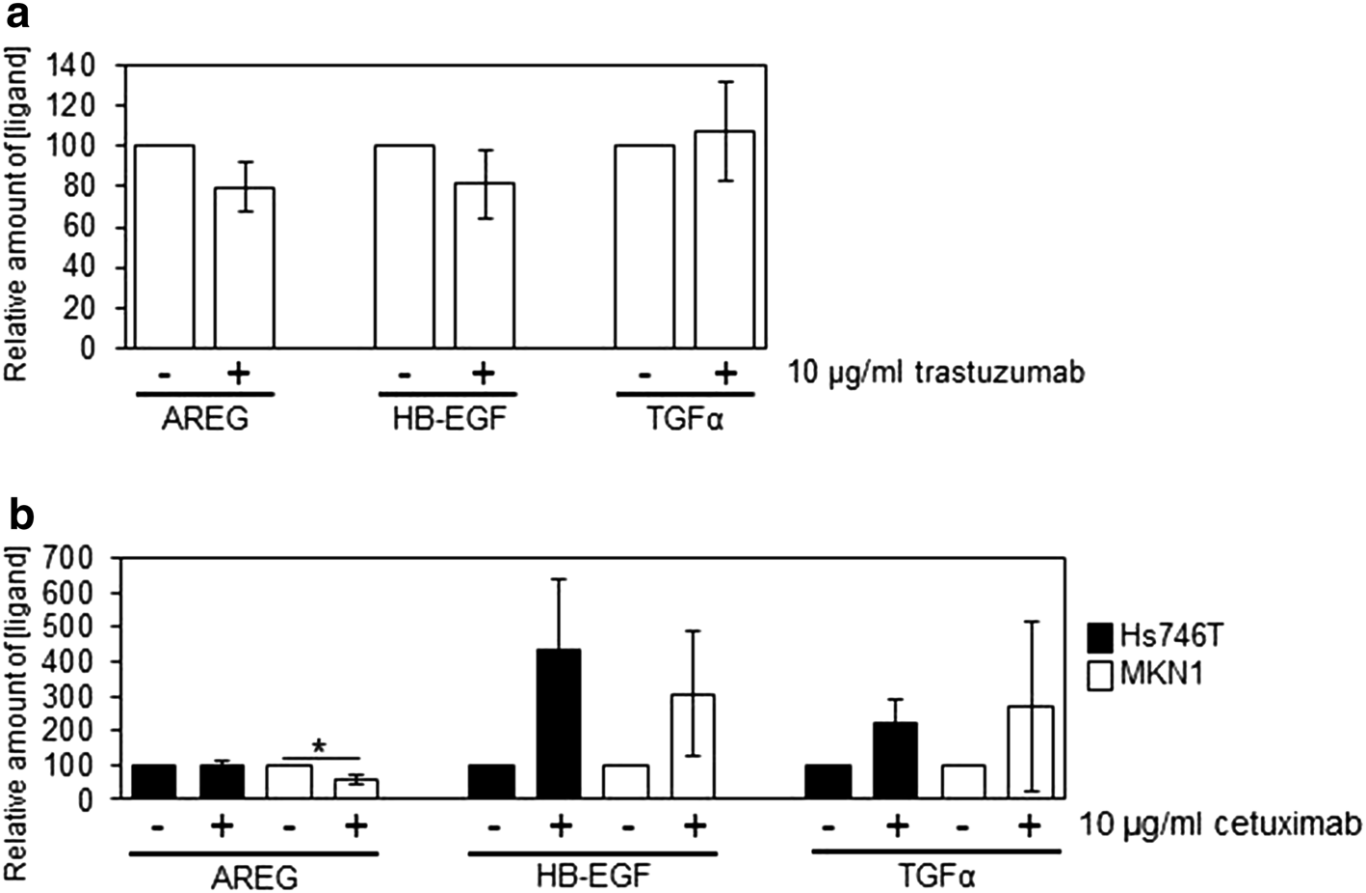
Effect of treatment with trastuzumab or cetuximab for 8 + 1 days on the HER receptor ligand secretion. Cells were treated for 8 days with trastuzumab (**a**; GSU cells) or cetuximab (**b**; MKN1 and Hs746T cells). Subsequently, the cells were seeded at defined densities and incubated for 24 h. Afterwards, the concentrations of AREG, HB-EGF and TGFα in the conditioned medium were determined using an ELISA. By non-significant trend, AREG secretion decreased after trastuzumab treatment. Cetuximab treatment significantly decreased AREG secretion of MKN1 cells and non-significantly increased TGFα and HB-EGF secretion in both, MKN1 and Hs746T cells. The mean value of three independent experiments is shown. *p* values at significance levels of ≤0.050 and ≤0.010 are indicated by (*) and (**), respectively

To investigate the effect of prolonged trastuzumab treatment on HER receptor ligand secretion, we treated GSU cells for 8 days with 10 µg/ml trastuzumab and measured the level of AREG, HB-EGF and TGFα in the cell culture supernatant. Although we did not observe any significant effects on ligand secretion, we found a decreasing trend in AREG secretion (*p* = 0.099). No relevant differences regarding the levels of HB-EGF and TGFα were detected (Fig. 6a).

In contrast, we observed after 8 days of cetuximab treatment an increase in HB-EGF and TGFα levels in MKN1 and Hs746T cells, although these effects were non-significant. Similar to trastuzumab treatment, application of cetuximab caused a significant decrease in AREG secretion in the cetuximab-sensitive cell line MKN1 (*p* = 0.043), while AREG levels in Hs746T were not altered (Fig. 6b).

### Effects of exogenous ligand application on trastuzumab sensitivity in gastric cancer cell lines

To investigate the effects of exogenous ligands on the trastuzumab sensitivity of gastric cancer cells, we performed a concomitant treatment of gastric cancer cell lines with AREG, EGF or HB-EGF and trastuzumab. We then measured the influence of this treatment on the metabolic activity of the cells via the WST-1 cell proliferation assay.

**Fig. 7:**
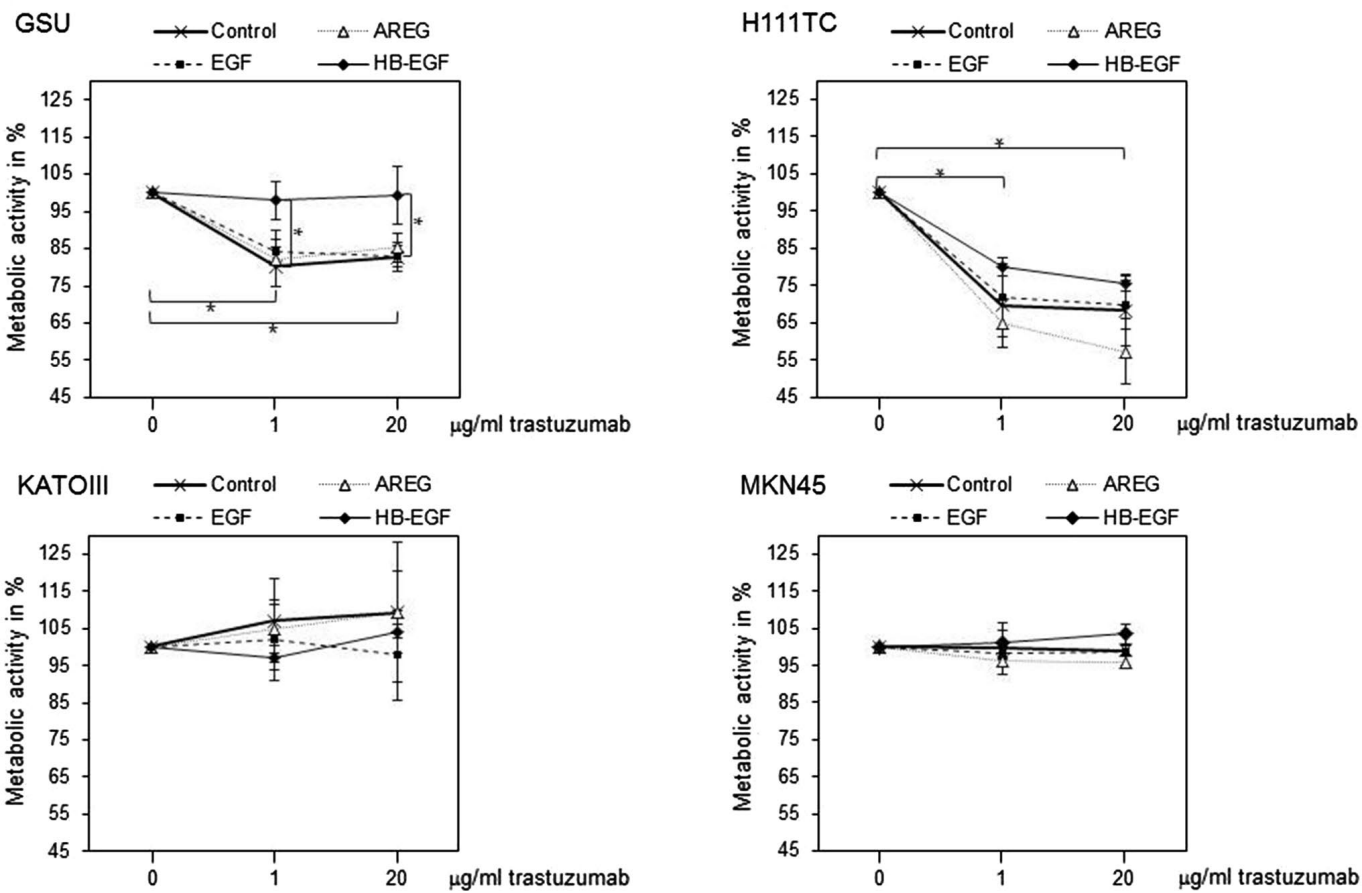
Effect of exogenous ligand application on trastuzumab sensitivity in gastric cancer cell lines. GSU, H111TC, KATOIII and MKN45 cells were treated for 3 days with trastuzumab alone (0, 1, 20 µg/ml) and/or different HER receptor ligands (AREG: 15 ng/ml; EGF: 0.1 ng/ml; HB-EGF: 0.4 ng/ml). The metabolic activity of the cells was measured using the WST-1 cell proliferation assay. In GSU cells, HB-EGF but not AREG and EGF was effective in rescuing the cells from trastuzumab treatment. In H111TC, a similar but not significant trend was observed. No effect of either ligand was detected for the trastuzumab-resistant cell lines KATOIII and MKN45. The mean value of three independent experiments is shown. For better readability only *p* values referring to the control are shown. For all significant *p* values, please refer to Table 2. *p* values at significance levels of ≤0.050 and ≤0.010 are indicated by (*) and (**), respectively

Ligand application had no effect on the proliferative activity of the trastuzumab-insensitive cell lines KATOIII and MKN45, as no differences between the treatments could be observed. In contrast, in the trastuzumab-sensitive cell lines GSU and H111TC, a combination of the ligands in addition to trastuzumab influenced the trastuzumab sensitivity in different ways. In GSU cells, trastuzumab application (20 µg/ml) inhibited the proliferative activity to 82.7% compared to the untreated control (*p* = 0.017). EGF and AREG were ineffective in rescuing the cells from growth inhibition, as there was no significant difference compared with the control. In contrast, HB-EGF completely neutralized the inhibitory effect of trastuzumab. For H111TC cells, HB-EGF application partially rescued the cells from trastuzumab inhibition; however, this effect was not significant. Additionally, we observed enhanced trastuzumab sensitivity of the cells after application of exogenous AREG (Fig. 7; Table 2).

**Table 2:** Effect of exogenous ligand application on trastuzumab sensitivity

Cell line	X	Y	*p* value
GSU	Untreated	1 µg/ml trastuzumab	0.023*
	Untreated	20 µg/ml trastuzumab	0.017*
	AREG	AREG/20 µg/ml trastuzumab	0.022*
	EGF	EGF/1 µg/ml trastuzumab	0.015*
	EGF	EGF/20 µg/ml trastuzumab	0.009*
	HB-EGF/1 µg/ml trastuzumab	1 µg/ml trastuzumab	0.013**
	HB-EGF/1 µg/ml trastuzumab	AREG/1 µg/ml trastuzumab	0.046**
	HB-EGF/1 µg/ml trastuzumab	EGF/1 µg/ml trastuzumab	0.021**
	HB-EGF/20 µg/ml trastuzumab	20 µg/ml trastuzumab	0.046**
H111TC	Untreated	1 µg/ml trastuzumab	0.043*
	Untreated	20 µg/ml trastuzumab	0.028*
	AREG	AREG/1 µg/ml trastuzumab	0.011*
	AREG	AREG/20 µg/ml trastuzumab	0.014*
	EGF	EGF/1 µg/ml trastuzumab	0.044*
	EGF	EGF/20 µg/ml trastuzumab	0.015*
	HB-EGF	HB-EGF/1 µg/ml trastuzumab	0.004*
	HB-EGF	HB-EGF/20 µg/ml trastuzumab	0.003*
	HB-EGF/1 µg/ml trastuzumab	AREG/1 µg/ml trastuzumab	0.044**
MKN45	AREG	AREG/1 µg/ml trastuzumab	0.022*
	HB-EGF/20 µg/ml trastuzumab	AREG/20 µg/ml trastuzumab	0.026**

All values were compared to the respective non-trastuzumab-treated reference by one-sample *t* test (*). Furthermore, the two-sided Welch *t* test was used for pairwise comparison of values treated with the same trastuzumab concentration and different ligand treatments (**)

Significant *p* values (refers to Fig. 7)

**Fig. 8:**
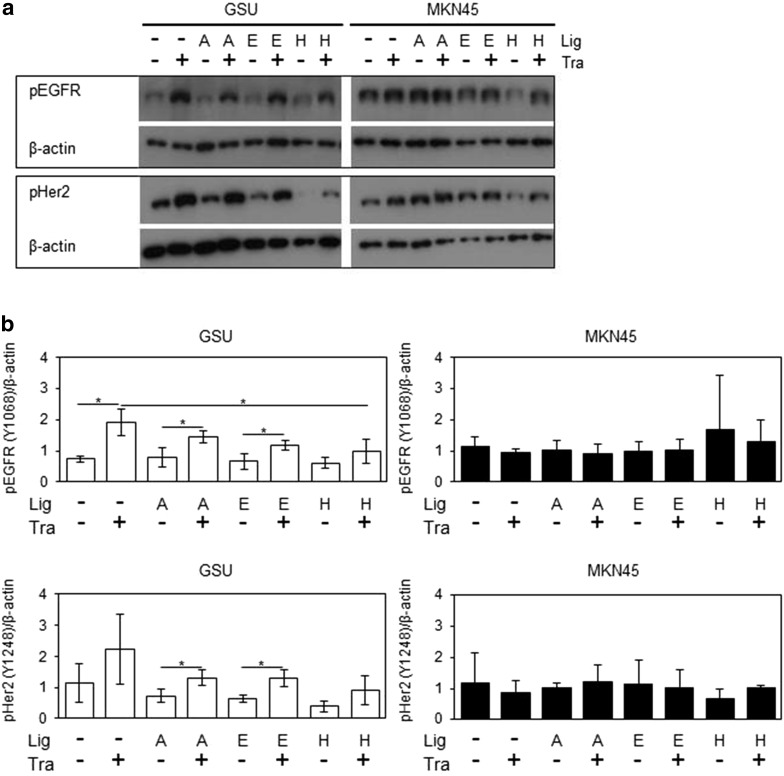
Effect of trastuzumab and concomitant ligand application on pEGFR and pHER2 levels in GSU and MKN45 cells. GSU and MKN45 cells were treated for 6 h with 10 µg/ml trastuzumab (Tra) and 15 ng/ml AREG (A) or 0.1 ng/ml EGF (E) or 0.4 ng/ml HB-EGF (H) or 0.75 ng/ml TGFα (T). Subsequently, pEGFR and pHER levels were determined by Western blot analysis.** a** Shows representative experiments;** b** shows results of densitometric measurements of three independent experiments. Treatment with trastuzumab induced the levels of both proteins in GSU cells but not in MKN45 cells. Concomitant application of the ligand suppressed this effect in GSU cells (significant suppression for pEGFR and HB-EGF). *p* values at significance levels of ≤0.050 and ≤0.010 are indicated by (*) and (**), respectively. Only relevant *p* values are shown. A list with all *p* values is shown in Online Source 6

Western blots were performed to investigate the effects of parallel treatment with trastuzumab and AREG, EGF, HB-EGF on pEGFR and pHER2 levels. For these experiments, a 6-h treatment was chosen. While no effect on the trastuzumab-insensitive cell line MKN45 could be observed, GSU showed an increase in pEGFR levels after the trastuzumab treatment in comparison with the untreated control (*p* = 0.038). Interestingly, concomitant application of either ligand suppressed this effect. However, only for HB-EGF was this suppression significant (*p* = 0.05). Regarding pHER2, similar, but non-significant patterns were observed (Fig. 8, Online Resource 8).

**Fig. 9:**
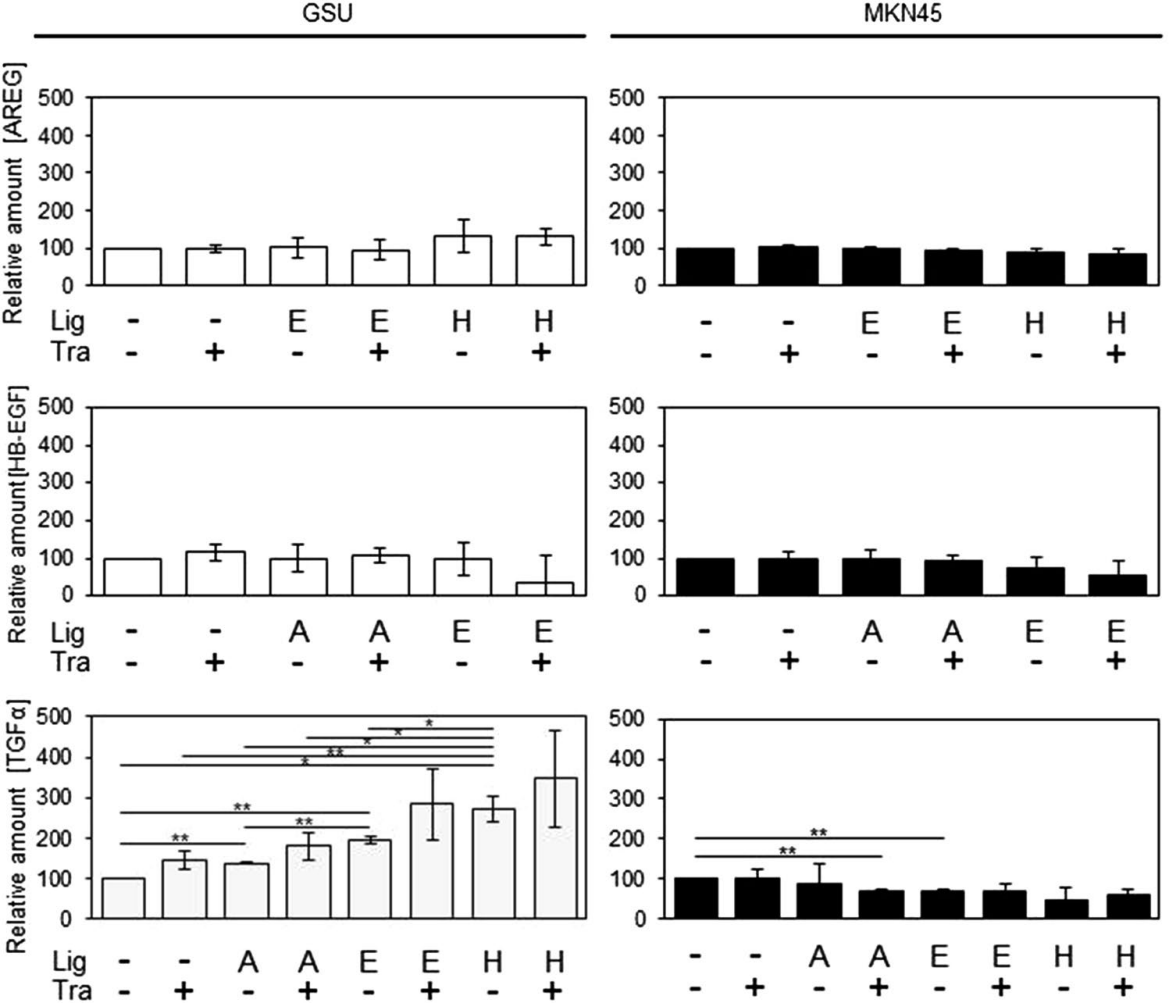
Effect of trastuzumab and concomitant ligand application on AREG, TGFα and HB-EGF levels in GSU and MKN45 cells. GSU and MKN45 cells were treated for 6 h with 10 µg/ml trastuzumab (Tra) and the HER receptor ligands (Lig) 15 ng/ml AREG (A) or 0.1 ng/ml EGF (E) or 0.4 ng/ml HB-EGF (H) or 0.75 ng/ml TGFα (T). Subsequently, concentrations of secreted AREG, HB-EGF and TGFα were determined in the conditioned cell culture medium using ELISAs. Application of each ligand caused a significant increase of TGFα secretion in GSU cells. For this, HB-EGF was more effective than EGF, and EGF was more effective than AREG. The mean value of three independent experiments is shown. *p* values at significance levels of ≤0.050 and ≤0.010 are indicated by (*) and (**), respectively

In addition, we measured the levels of the ligands AREG, HB-EGF and TGFα in the conditioned medium of GSU and MKN45 cells, following treatment for 6 h with trastuzumab and/or exogenous ligand (AREG, EGF, HB-EGF). Levels of HB-EGF and AREG showed no significant change with any of the treatments, but application of each ligand caused a significantly increased secretion of TGFα in GSU cells. Regarding this effect, HB-EGF was more effective than EGF, and EGF was more effective than AREG. Interestingly, in the trastuzumab-insensitive cell line MKN45, a significant decrease of TGFα was observed after treatment with AREG/trastuzumab and EGF (Fig. 9).

### Effects of exogenous ligand application on cetuximab sensitivity in gastric cancer cell lines

We investigated the effects of different concentrations of AREG, EGF and HB-EGF on the cetuximab sensitivity of the sensitive cell line MKN1 and the resistant cell line Hs746T. We found both, HB-EGF and EGF, but not AREG, to be effective in rescuing MKN1 from cetuximab inhibition. Surprisingly, we detected a minor inhibition in the cetuximab-resistant cell line Hs746T by cetuximab application; however, exogenous ligand application had no additional effect (Online Resource 9).

### Prognostic relevance of HER receptor ligands in gastric cancer as described in the literature

To investigate the relevance of HER receptor ligands in gastric tumours, a literature research was performed. As shown in Table 3, the expression of AREG, EGF, HB-EGF and TGFα in gastric tumours and body fluids of gastric cancer patients has been analysed in multiple studies. The majority of the articles investigated the expression of EGF and TGFα.

**Table 3:** Overview of published studies examinating the expression of AREG, EGF, HB-EGF and TGFα in gastric cancer patients

References	Ligand	Detected	Method	Origin of human samples	Number of GC patients	Treatment	Observations
Han et al. (2009)	AREG, EGF, TGFα	Protein	ELISA	Serum of AGC patients	38	Cetuximab combined with modified FOLFOX6	Low EGF and TGFα serum levels: associated with higher response rate (*p* = 0.01/0.03) AREG serum levels: not predictive for response rate, median TTP, median OS All patients with EGFR expression and low EGF and TGFα levels: response to therapy (*p* < 0.001), longer OS and TTP (after adjusting for clinical factors) Eight patients with follow-up serum samples and disease progression: EGF or TGFα elevation in seven patients
Yoshida et al. (2012)	Pro-AREG	Protein	IHC	Unresectable AGC samples	46	S1-based regimen	34.8% AREG (+), 65.2% AREG (−) MS: AREG-positive 311 d, AREG-negative 387 d (*p* = 0.046) No further significant observations
Saeki et al. (1994) Saeki	AREG, TGFα	Protein	IHC	GC samples, adjacent intestinal metaplasia, adjacent uninvolved mucosa	37		AREG (+): 51% of GC samples; 26% of intestinal metaplasia samples; 21% of normal mucosa samples TGFα (+): 57% of GC samples; 17% of intestinal metaplasia samples; 0% of normal mucosa samples
Yasumoto et al. (2011)	AREG, EGF, HB-EGF, TGFα	Protein	ELISA	Malignant ascites of GC patients, non-malignant ascites	20	Gastrectomy of previously untreated GC patients	Enhanced levels of HB-EGF, AREG in malignant ascites compared to non-malignant ascites EGF, TGFα barely detectable in malignant and non-malignant ascites
Kitadai et al. (1993)	AREG	mRNA Protein	NB ICC	GC samples, corresponding normal mucosa	32	surgery	AREG mRNA detectable in all samples (tumour, normal mucosa) In 62.5% of tumours: increased AREG mRNA expression compared to normal mucosa No correlation of AREG mRNA levels with histological types, staging
Nielsen et al. (2014)	AREG, EGF, HB-EGF, TGFα^a^	mRNA	qRT-PCR	GC samples, GEJ-, oesophagus-cancer samples, adjacent normal mucosa	18 (20?)	surgery	EGF mRNA hardly detectable in tumour and normal tissue Up-regulation of all AREG, HB-EGF, TGFα
Zhang et al. (2014)	AREG, TGFα, EGF^b^	Protein	RIA, Sandwich-ELISA	Serum of GC/GEJ-cancer patients	29 (GC) 23 (GEJ)	Cetuximab + cisplatin + capecitabine	Patients with higher TGFα levels: longer PFS, longer OS (*p* = 0.003, 0.008) Patients with partial/full recovery: higher TGFα levels than SD/PD group (*p* = 0.025) Patients wither higher EGF levels: longer OS (*p* = 0.061) No correlation between AREG and efficacy
Naef et al. (1996)	AREG, HB-EGF^a^	mRNA, protein	NB RT-PCR IHC	GC samples, corresponding mucosa	12	Surgery	4.7-fold increase in HB-EGF transcript in GC samples compared to normal mucosa (*p* < 0.006) AREG transcript: not detectable via NB; in RT-PCR: detectable in all controls, 5 of 8 GC samples (62.5%) IHC: 5 of 7 GC samples (71.4%) HB-EGF (+)
Shimura et al. (2012)	Cytoplasmic domain of proHB-EGF (HB-EGF-C)	Protein	IHC	GC samples	96		45.8% HB-EGF-C positive; increase in expression, nuclear localization in pT3, pT4 compared to pT1, pT2 (*p* < 0.001)
Murayama et al. (2002)	HB-EGF, proHB-EGF	Protein mRNA	IHC, WB, NB, in situ hybridization	GC samples, corresponding mucosa	66	Surgery, no previous chemo-, radiotherapy	mRNA detectable in all tumour samples in NB; in situ hybridization: mRNA expression in intestinal tumour cells proHB-EGF protein detectable in 60.6% of GC samples proHB-EGF protein expression more frequent in advanced T stages (*p* < 0.01) proHB-EGF protein expression more frequent in intestinal type versus diffuse type (*p* < 0.001) No significant association of proHB-EGF protein expression with age, sex, lymph node status, pathological status
Hirata et al. (2009)	HB-EGF	Protein	IHC	AGC samples with adjacent non-neoplastic mucosa	100	Surgical resection	HB-EGF (+): 48% of the cases HB-EGF (−): 52% of the cases
Chung et al. (2015)	Soluble HB-EGF	Protein	ELISA	Serum of AGC, EGC patients, high risk patients, healthy controls	37 EGC 30 AGC	NR	Increase of sHB-EGF along the GC carcinogenic sequence (*p* < 0.001) *Significantly elevated sHB*-*EGF levels* In AGC patients compared to the other groups (*p* < 0.001) In EGC patients compared to high risk patients (*p* = 0.049) In EGC patients compared to control group (*p* = 0.006) In cancer groups compared to non-cancer groups (*p* < 0.001) No further significant differences between groups Serum sHB-EGF significantly associated with age, T, N, M, overall stage, tumour size (*p* = 0.001/<0.001/= 0.001/= 0.030/<0.001/= 0.048) Serum sHB-EGF rated as accurate diagnostic biomarker for prediction of GC
Suganuma et al. (2003)	HB-EGF	mRNA	Oligo-nucleotide microarray	AGC samples with corresponding normal tissue	35	Surgery, chemo-therapy	HB-EGF expression up-regulated in cisplatin- and 5-FU-resistant tumours
Onda et al. (1990)	EGF	Protein	IHC	EGC, AGC samples, non-cancerous tissue, dissected lymph nodes	185	Gastrectomy	EGF(+): 55% AGC, 19% EGC (*p* < 0.01) EGF(+): 37% non-scirrhous GC, 69% scirrhous GC (*p* < 0.05) Correlation with lymph node metastases: EGC *p* < 0.005, AGC *p* < 0.05 5-year survival worse in EGF (+) patients versus EGF (−) patients (*p* < 0.05) in EGC and AGC patients EGF detected more often in invasive GC No difference between intestinal and diffuse GC type
Czyzewska et al. (2009)	EGF	Protein	IHC	AGC samples, metastatic lymph nodes	55	Surgery	Association between EGF expression in primary tumour and lymph node metastasis (*p* = 0.000) High EGF levels in tumour main mass associated with longer survival In patient group with low EGF expression: increased mortality after 11–23 month (*p* = 0.03) No further significant correlations
Park do et al. (2014)	EGF^b^	Protein	ELISA	Serum of GC/GEJ-cancer patients	147	Gastrectomy, esophagogastrectomy	*In pre*-*treatment blood samples* High EGF levels associated with poorly/undifferentiated differentiated tumours (*p* = 0.020)
Docea et al. (2013)	EGF	Protein	IHC	gastric intestinal tumours	25	Surgery, no adjuvant treatment	EGF detected in 88% of tumour samples higher EGF score associated to low-grade tumours (*p* = 0.010) No further significant correlations with clinicomorphological features
Pryczynicz et al. (2009)	EGF	Protein	IHC	GC samples	55	NR	EGF (−): 54.5% EGF (+): 45.5% No significant correlation with degree of *H. pylori* infection
Rajcevic et al. (2001)	EGF^a^	mRNA	Fluorescent multiplex RT-PCR	GC samples, peripheral normal mucosa	29	NR	Level of EGF overexpression: None: 39%; <5×: 50%; 5–10×: 11%
Yasui et al. (1988)	EGF	Protein	IHC	EGC, AGC samples	156	Surgical resection	EGC: EGF (−) AGC: EGF (+) 29.2% of the cases Correlation EGF (+) with tumour depth (*p* < 0.05) Higher incidence of EGF (+) in well than in poorly differentiated tumours (*p* < 0.05) Higher incidence of EGF (+) in metastatic than in primary tumours (*p* < 0.05) Patients with EGF (+) tumours: poorer prognosis
Tahara et al. (1986)	EGF	Protein	IHC, RIA	AGC, EGC, scirrhous GC samples	210	Surgical resection	EGC: EGF (−) AGC: EGF (+) 21.2% of cases Scirrhous GC: EGF (+) 33.3% of cases EGF (+): significantly higher in well differentiated than in poorly differentiated adenocarcinoma EGF (+): significantly higher in poorly differentiated scirrhous GC than in poorly differentiated adenocarcinoma EGF (+) patients (without scirrhous GC): much worse prognosis
Hirayama et al. (1992)	EGF^b^	Protein	IHC	EGC samples (penetrating and non-penetrating type), AGC samples	46	NR	EGC (non-penetrating): 11.1% EGF (+) EGC (penetrating): 42.9% EGF (+)* AGC: 50% EGF (+)* * *p* < 0.05 to EGC (non-penetrating) No significant correlation between vessel invasion and EGF-positive rates Significant higher EGF-positive rate when lesion with large amount of intestinal connective tissue for EGC (penetrating) and AGC
Yoshiyuki et al. (1990)	EGF	Protein	IHC	Primary GC samples (*n* = 24), lymph node metastases (*n* = 8)	32	Surgery	EGF (+): 50% of the cases (primary GC and lymph node metastases)
Oda et al. (1990)	EGF	Protein	IHC	GC samples	36	Surgical resection	EGF (+): 30% of the cases; 28% of diploid tumours, 33% of aneuploidy tumours, no significant difference
Dias et al. (2011)	EGF, TGFα	Protein	RIA	Gastric juice of patients with *HP*-induced chronic atrophic gastritis, intestinal metaplasia, gastric adenocarcinoma, HP-negative controls with non-ulcer dyspepsia	9	NR	EGF levels in GC patients > fourfold elevated compared to controls (*p* < 0.001), threefold elevated compared to chronic atrophic gastritis patients (*p* < 0.05) TGFα levels in GC patients: half the value compared to controls and chronic atrophic gastritis patients (*p* < 0.05)
Dragovich et al. (2006)	EGF, TGFα	Protein	ELISA IHC (TGFα)	Plasma of GC/GEJ-cancer patients, tumour samples	43 (GEJ) 25 (GC)	Erlotinib	EGF levels in plasma: no difference between responders and non-responders TGFα: hardly detectable in plasma; IHC: 74% of samples TGFα (+); not predictive for clinical outcome
Aoyagi et al. (2001)	EGF, TGFα	Protein	IHC	GC samples (superspreading type (su.sp), penetrating type (pen.)	151 su.sp.: 39 pen: 11	Gastrectomy	Intramucosal GC: EGF (+) 14.7%, TGFα (+) 35.3% Submucosal GC: EGF (+) 15.8%, TGFα (+) 47.4% Small submucosal GC: EGF(+) 20.0%, TGFα (+) 60.0% Su.sp.: EGF(+) 15.3%, TGFα (+) 33.3% Pen: EGF(+) 54.5%, TGFα (+) 63.6% Pen significantly higher rate of EGF (+) than su.sp. (*p* < 0.05) TGFα (+): correlation with tumour size: tumour size - 20 mm: 20.5%, tumour size 20–50 mm: 56.5% (*p* < 0.01)
Borlinghaus et al. (1993)	EGF, TGFα	Protein	RIA	GC samples, surrounding mucosa	11	Surgical resection	EGF expression in 3 out of 10 tumours TGFα expression in 11 GC samples TGFα expression higher in normal mucosa than in malign tissue
Livingstone et al. (1995)	EGF, TGFα	Protein	IHC	GC samples of 33 European and 40 Japanese patients	73	Surgical resection	EGF(+): 55% (Japanese), 58% (European); *p* = NS TGFα (+): 54% (Japanese), 72% (European); *p* = NS
Yoshida et al. (1990)	EGF, TGFα	mRNA		GC samples, corresponding normal mucosa	15	Surgery	TGFα mRNA: detectable in all tumour samples and corresponding normal tissue; EGF mRNA detected in 33.3% of GC samples
Sugiyama et al. (1989)	EGF	Protein	IHC	GC samples	222	Gastrectomy	EGF (+): 29% of GC samples
Kim et al. (2013)	TGFα	Protein	IHC	GC samples; controls: chronic gastritis, metaplasia, low-grade epithelial dysplasia	206	Total, subtotal gastrectomy	TGFα (+): 26.3% TGFα expression higher in GC than in controls (*p* < 0.05) TGFα expression rate higher in intestinal than in diffuse tumours (*p* < 0.05)
Chuang et al. (1994)	EGF, TGFα	Protein	RIA	Urine of patients with cancers of the digestive tract, healthy controls	15		EGF (+): 66.7% of GC samples EGF/TGFα (+): 33.3% of GC samples Utility as diagnostic marker: EGF and TGFα show high specificity (100%), EGF shows high sensitivity (100%)
Fanelli et al. (2012)	TGFα	Protein	IHC	GC samples	137	Total, subtotal gastrectomy	High TGFα expression correlated with poor OS No significant correlation observed for staging, classification
Celikel et al. (2007)	TGFα	Protein	IHC	GC samples	101	Total, subtotal gastrectomy	TGFα (+): 42.6% of GC cases Association with lymph node involvement (*p* = 0.014), perineural invasion (*p* = 0.016) Patients with TGFα (−) tumours: significantly longer survival (*p* = 0.002)
Espinoza et al. (2004)	TGFα	Protein	IHC	GC samples	100	Total, partial gastrectomy, no pre-surgical adjuvant treatment	TGFα (+): 51% of GC cases Positive correlation with lymph node metastasis (*p* = 0.001), TNM stage (*p* = 0.036)
Konturek et al. (2001)	TGFα^b^	mRNA Protein	RT-PCR, WB	GC samples, adjacent and remote intact mucosa, biopsies of normal controls	25	NR	mRNA: TGFα (+): 48% of GC samples, 24% of adjacent/normal intact mucosa samples, in densitometric measurement: twofold increase of TGFα mRNA expression in GC tissue compared to adjacent mucosa Protein: increased TGFα expression: 35% of GC samples
Choi et al. (1999)	TGFα	Protein	ELISA	Serum of GC patients, healthy controls	40	None: *n* = 36 Surgery: *n* = 4	Mean TGFα serum levels: 104 ± 235 pg/ml (patients), 22 ± 16 pg/ml (healthy controls), *p* = 0.03 no association with clinicopathologic characteristics TGFα (+): associated with poorly differentiated tumours (*p* = 0.060)
Takita et al. (1998)	TGFα	Protein	IHC	EGC, AGC and AGC with hepatic metastasis samples	82	Surgery, no prior chemo-, radiotherapy	In 17 paired samples: TGFα (+): 13 primary tumours and 15 hepatic metastasis
Moskal et al. (1995)	TGFα	Protein	RIA	Serum of GI patients, healthy controls	11	None: *n* = 2 OngT: *n* = 6 PostT: *n* = 3	TGFα concentration: 179–375 pg/ml (mean: 231 pg/ml) Significant higher than in healthy controls (*p* < 0.0001)
Muller and Borchard (1992)	TGFα	Protein	IHC	GC samples, normal mucosa	120	NR	TGFα (+): 60% of tumours, 36% of normal mucosa samples EGC: 50% TGFα (+); AGC: 63% TGFα (+) No significant correlation with clinical and pathologic characteristics, no significant correlation with prognosis
Nasim et al. (1992)	TGFα	Protein	IHC	GC samples, normal mucosa, intestinal metaplasia, dysplasia	24	NR	Diffuse GC: 30% weak cytoplasmic staining Intestinal GC: 93% strong cytoplasmic staining
Bennett et al. (1989)	TGFα	mRNA	Dot Blot hybridization NB	AGC, lymphoma, benign ulceration samples, adjacent non-malignant tissue for 16 GC cases	26	Gastrectomy	15 of 18 GC tumours and 10 of 16 adjacent non-malignant samples: TGFα mRNA expression In most cases higher TGFα mRNA expression in tumour than in non-malignant tissue 9 GC tumours: very high TGFα mRNA expression
Beauchamp et al. (1989)	TGFα	mRNA Protein	NH RIA	GC samples, adjacent uninvolved mucosa	10	NR	In 6 patients: increase in TGFα expression in tumour compared to mucosa (5 patients: well or moderately differentiated tumours) In 4 patients: no difference (poorly differentiated tumours) 6 samples: TGFα protein levels detected via RIA, all TGFα (+)
Wilgenbus et al. (1992)	TGFα	Protein	IHC	25 GC samples + 1 patient with acanthosis nigricans (tumour and skin samples)	26	Gastrectomy NR	Patient with acanthosis nigricans: TGFα (+) tumour, weak staining in skin biopsies Other GC samples: 1 of 25 TGFα (+)

GC = gastric cancer; AGC = advanced gastric cancer; EGC = early gastric cancer; GEJ = gastroesophageal junction; GI = gastrointestinal cancer; (+) = positive; (−) = negative; NS = non-significant; *HP* *=* *Helicobacter pylori*; ICC = immunocytochemistry; IHC = immunohistochemistry; RIA = radioimmunoassay; WB = Western blot; NB = northern blot; MS = median survival; PFS = progression-free survival; OS = overall survival; SD = stable disease; PD = progressive disease; d = days; NR = not reported; OngT = ongoing treatment; PostT = posttreatment

^a^Additional HER ligands detected in the same study

^b^Additional growth factors detected in the same study

EGF protein expression was found in up to 88% of the gastric tumour samples, with most studies reporting EGF positivity in 29–58% of the cases (Aoyagi et al. 2001; Borlinghaus et al. 1993; Docea et al. 2013; Hirayama et al. 1992; Livingstone et al. 1995; Oda et al. 1990; Onda et al. 1990; Pryczynicz et al. 2009; Sugiyama et al. 1989; Tahara et al. 1986; Yasui et al. 1988; Yoshiyuki et al. 1990). EGF was detected in the serum, plasma, urine and gastric juice of patients with gastric cancer, while analysis of the malignant ascites revealed only very low EGF levels (Chuang et al. 1994; Dias et al. 2011; Dragovich et al. 2006; Han et al. 2009; Park do et al. 2014; Yasumoto et al. 2011; Zhang et al. 2014). Several studies indicated an association with advanced disease, metastatic disease and poor prognosis (Czyzewska et al. 2009; Hirayama et al. 1992; Onda et al. 1990; Tahara et al. 1986; Yasui et al. 1988), while in early gastric cancers, per trend a smaller percentage of EGF-positive tumours was reported (Aoyagi et al. 2001; Hirayama et al. 1992; Onda et al. 1990; Tahara et al. 1986; Yasui et al. 1988). Regarding the predictive value of EGF expression for EGFR-targeted therapies, the results are completely contradictory; in patients treated with cetuximab combined with modified FOLFOX6, low EGF levels were associated with a higher response rate (Han et al. 2009). In contrast, patients with high EGF serum levels treated with cetuximab, cisplatin and capecitabine displayed a longer overall survival (Zhang et al. 2014). In addition, EGF plasma levels were not found to be predictive for therapy response in patients treated with erlotinib (Dragovich et al. 2006).

Similarly inconsistent findings concerning EGFR-targeted therapies were reported for TGFα (Dragovich et al. 2006; Han et al. 2009; Zhang et al. 2014). Expression of the TGFα protein was reported in most studies in between 35 and 74% of gastric tumours (Celikel et al. 2007; Dragovich et al. 2006; Espinoza et al. 2004; Konturek et al. 2001; Livingstone et al. 1995; Muller and Borchard 1992; Saeki et al. 1994). Regarding the general prognostic value of TGFα, data are quite inconsistent; high TGFα levels in the tumour were correlated with lymph node metastasis, poor overall survival, advanced TNM stage and tumour size in some studies (Aoyagi et al. 2001; Celikel et al. 2007; Espinoza et al. 2004; Fanelli et al. 2012). On the other hand, no correlation with clinicopathologic features and prognosis was reported in another study (Muller and Borchard 1992). Furthermore, serum TGFα levels showed no correlation with clinicopathologic characteristics (Choi et al. 1999).

Only a few studies have investigated the expression of AREG in gastric cancer. Positive protein staining was reported for 34.8% (pro-AREG) and 51% (AREG) of the tumours (Saeki et al. 1994; Yoshida et al. 2012). AREG mRNA was detected in malignant as well as in non-malignant tissue (Kitadai et al. 1993; Naef et al. 1996; Nielsen et al. 2014). However, an increase of AREG mRNA expression in tumour tissue was reported twice (Kitadai et al. 1993; Nielsen et al. 2014). One study found no correlation between AREG mRNA expression and staging or histological tumour types, and another study reported a significantly shorter median survival in patients with pro-AREG-positive tumours (Kitadai et al. 1993; Yoshida et al. 2012). In addition, high AREG concentrations were found in the malignant ascites of gastric cancer patients (Yasumoto et al. 2011). AREG serum levels were not predictive for response rate, median time to progression or median overall survival (Han et al. 2009).

Regarding HB-EGF, data are highly consistent and indicate an association with advanced disease: levels of soluble HB-EGF were found to be elevated in the serum of advanced gastric cancer patients (Chung et al. 2015) as well as in malignant ascites of gastric cancer patients (Yasumoto et al. 2011). Furthermore, increased expression of proHB-EGF and of the cytoplasmic domain of HB-EGF in advanced tumour stages was reported (Murayama et al. 2002; Shimura et al. 2012). Interestingly, there is additional evidence that HB-EGF might be a resistance factor against 5-FU- and cisplatin-based chemotherapies (Suganuma et al. 2003).

## Discussion

The aim of this study was to investigate the involvement of several HER receptor ligands in the response of a panel of 11 gastric cancer cell lines to cetuximab and trastuzumab. Seven of these cell lines had been characterized regarding their cetuximab sensitivity in studies published previously by our group (Heindl et al. 2012; Kneissl et al. 2012). Four additional cell lines were investigated in the present study, and we found three cell lines, GSU, H111TC and MKN7, to be cetuximab-sensitive, while HGC-27 showed a cetuximab-resistant phenotype. Interestingly, we identified an activating *KRAS* mutation in GSU cells. Such mutations are known resistance factors against cetuximab in colorectal cancer (Karapetis et al. 2008; Lievre et al. 2006). In a previous study, we associated the cetuximab resistance of the gastric cancer cell line AGS with an activating *KRAS* mutation (Kneissl et al. 2012). However, this new finding indicates that such mutations are not necessarily associated with cetuximab resistance in gastric cancer.

Only 2 out of 11 cell lines (18.18%) were sensitive to trastuzumab treatment. To our knowledge, these two cell lines, GSU and H111TC, have not been previously described to be trastuzumab sensitive. Mutations in the *PIK3CA* gene were recently associated with reduced response of breast cancer patients to trastuzumab and/or lapatinib neoadjuvant therapy (Majewski et al. 2015). In line with these data, we found no such mutation in hot spot regions for both sensitive cell lines, while four of the insensitive cell lines were described to harbour mutations (Mita et al. 2009; Zhou et al. 2011). For most resistant cell lines, the trastuzumab sensitivity had been investigated in prior studies. Cell lines we identified as resistant had been described to be trastuzumab resistant or mildly sensitive in other publications (Liu et al. 2015; Tomioka et al. 2012; Wainberg et al. 2010). The minor differences between prior studies and our findings are in the expected range regarding results from cell viability assays. In our study, GSU and H111TC showed only a moderate sensitivity to the monoclonal antibody with a growth inhibition of approximately 20% compared to the untreated control. However, we were able to enhance this effect by concomitant application of chemotherapeutics. These findings reflect the situation in gastric cancer patients, as trastuzumab has been combined with chemotherapy in the pivotal studies and the effect that results from addition of trastuzumab is only moderate (Bang et al. 2010; Okines et al. 2010).

On a molecular level, GSU cells showed induction of pHER2 (Y1248) expression after treatment with trastuzumab for 6 h, while no effect was observed for the resistant cell line MKN45. A similar effect was observed after 8 days of trastuzumab treatment for GSU and H111TC cells; however, the effect was not strong enough to persist in the densitometric measurement. These findings are in line with results obtained in breast cancer cells, where sensitive cell lines showed enhanced expression of pHER2 (Y1248) upon trastuzumab therapy (Diermeier et al. 2005; Dokmanovic et al. 2014). A similar observation was reported for NCI-N87 gastric carcinoma cells, although another publication found no such effect (Leto et al. 2015; Yamashita-Kashima et al. 2011).

Changes in the expression profile of the HER receptors were small following treatment with trastuzumab and cetuximab for 8 days. However, trastuzumab treatment showed more prominent effects, as HER2 levels were significantly downregulated in 4 cell lines, and pHER3 levels were found to be downregulated in two cell lines. We were not able to correlate these alterations to trastuzumab sensitivity or resistance. In contrast, we were surprised by the negligible impact of the cetuximab treatment, as we found only two significant increases in HER2 levels and one in pHER2 levels. On pEGFR level, we detected a complete absence of any effect, a finding that corresponded to results published recently by our group (Kneissl et al. 2012). It is arguable whether the weaker inhibitory effects of cetuximab on gastric cancer in comparison with trastuzumab are based on these differences.

The main focus of this study was the involvement of HER receptor ligands in the sensitivity of gastric cancer cell lines to trastuzumab and cetuximab. For our analyses, we concentrated mainly on AREG, HB-EGF and TGFα, as previous results from our group had shown the absence of EGF secretion in gastric cancer cell lines (Kneissl et al. 2012).

Although there is no known ligand binding to HER2, several studies have discussed the involvement of the HER receptor ligand system in the resistance to HER2-targeted therapies. Elevated levels of TGFα in the serum of breast cancer patients were associated with a poor response to lapatinib/capecitabine (Rhee et al. 2011). Furthermore, TGFα expression was found to be induced by trastuzumab application in breast cancer patients (Valabrega et al. 2005). In addition, in patients with metastatic HER2-positive breast cancer receiving trastuzumab plus taxane, the progression-free survival was significantly shortened in patients with high serum concentrations of AREG (Kim et al. 2015). In cell culture, breast cancer cells with secondary resistance to trastuzumab displayed an up-regulation of mRNA expression for EGF, TGFα, HB-EGF and heregulin, while the expression of AREG mRNA was downregulated and the expression of epiregulin and betacellulin mRNA was unchanged. Levels of secreted TGFα in the cell culture medium were found to be increased as well. Furthermore, co-treatment of BT-474 breast carcinoma cells with TGFα and trastuzumab neutralized the inhibition of the cell growth by trastuzumab (Ritter et al. 2007). Additionally, HB-EGF expression was linked to trastuzumab resistance in breast cancer cells (Yotsumoto et al. 2010). In our study, only secretion of TGFα increased in GSU cells after 6 h of trastuzumab treatment; however, this effect was not significant. After 8 days of trastuzumab application, we found a non-significant decrease of AREG secretion, while HB-EGF and TGFα were unaltered. The reasons for the differences in the findings mentioned above are likely due not only to different tumour entities but also to differences in the experimental setting, as Ritter et al. used a xenograft-based approach with a substantially longer period of trastuzumab treatment to isolate cancer cells with secondary trastuzumab resistance. We were not able to find any correlation between the levels of secreted TGFα and the sensitivity to trastuzumab. However, regarding HB-EGF, the two sensitive cell lines GSU and H111TC showed only minor HB-EGF secretion and the three cell lines displaying the highest amounts of HB-EGF in the conditioned medium, Hs746T, MKN7 and MKN28, were all trastuzumab resistant. Additionally, exogenous HB-EGF application neutralized the growth inhibition by trastuzumab completely in GSU cells and partially in H111TC cells. In GSU cells, this effect was accompanied by a block of the trastuzumab-driven induction of pHER2 (Y1248) expression. These findings indicate that HB-EGF is effective in neutralizing the effects of trastuzumab in gastric cancer cells.

The influence of the HER receptor ligand system on the responsiveness of solid tumour to cetuximab has been topic of numerous studies published in the last years, although only few studies dealt with the situation in gastric cancer.

In colorectal cancer patients, AREG expression in particular has been repeatedly associated with an enhanced responsiveness to cetuximab, especially for *KRAS* wild-type tumours (Baker et al. 2011; Jacobs et al. 2009; Khambata-Ford et al. 2007; Pentheroudakis et al. 2013; Yoshida et al. 2013). One study reported no correlation between AREG expression and outcome, while results published recently revealed that colorectal cancer patients displaying increased AREG plasma levels after the first application of cetuximab showed a poor clinical outcome (Kuramochi et al. 2012; Loupakis et al. 2014).

Regarding gastric cancer, no correlation was found between AREG serum levels and the response to cetuximab in combination with a modified FOLFOX6 regimen (Han et al. 2009). However, results published recently by our group identified AREG secretion in combination with other factors as a positive predictor of cetuximab response in gastric cancer cell lines (Kneissl et al. 2012). In the present study, we characterized four additional cell lines for cetuximab sensitivity and AREG secretion. These latest findings are in line with prior results, as we found all three cetuximab-sensitive cell lines—GSU, H111TC and MKN7—secreted AREG. Interestingly, GSU and H111TC secreted much higher amounts than MKN7, while MKN7 is far less sensitive to cetuximab than GSU and H111TC. We furthermore found AREG secretion to be significantly downregulated after 8 days of cetuximab treatment in the cetuximab-sensitive cell line MKN1 but not in cetuximab-resistant Hs746T cells.

For TGFα, results from the published literature have been inconsistent. In colorectal cancer cells and cell lines from tumours of the head and neck, overexpression and secretion of TGFα were associated with cetuximab resistance (Hobor et al. 2014; Saki et al. 2013; Troiani et al. 2013). However, patient-based data showed a different picture: in metastatic colorectal cancer patients treated with cetuximab, the presence of TGFα positivity correlated with a better outcome (Yoshida et al. 2013). In six colorectal cancer patients with wild-type *KRAS*, only serum TGFα levels consistently increased during cetuximab treatment, but such a consistent effect was not observed for other ligands (Mutsaers et al. 2009). In contrast, another study found no correlation of TGFα serum levels and clinical outcome in KRAS wild-type metastatic colorectal cancer patients treated with panitumumab or cetuximab (Takahashi et al. 2014). Moreover, several studies consistently found no association between TGFα mRNA expression and clinical outcome of cetuximab-based therapies in colorectal cancer patients (Baker et al. 2011; Cushman et al. 2015; Khambata-Ford et al. 2007; Pentheroudakis et al. 2013). However, in patients with rectal cancer, TGFα levels, but not EGF levels, increased during cetuximab treatment, and this increase was correlated with T downstaging (Debucquoy et al. 2009). In contrast, in patients suffering from tumours of the head and neck, there was no correlation between TGFα expression and efficacy of a cetuximab/bevacizumab regimen, and also TGFα levels significantly increased upon treatment (Argiris et al. 2013). In gastric cancer, data are contradictory, as one study associated higher TGFα serum levels with a better response to cetuximab, longer progression-free survival and longer overall survival, while another publication reported a correlation between low TGFα serum levels and a higher response rate to cetuximab in combination with modified FOLFOX6 (Han et al. 2009; Zhang et al. 2014). In our cell-based system, there was no correlation between TGFα secretion status and cetuximab sensitivity of the cell lines. However, after 8 days of cetuximab treatment, we detected a non-significant increase in secreted TGFα levels in the cetuximab-sensitive cell line MKN1 and the cetuximab-resistant cell line Hs746T. Further analyses are needed to clarify the role of TGFα in response to cetuximab.

Only a few studies have investigated the association between the cetuximab response and HB-EGF expression, and the data are inconsistent: HB-EGF expression was positively correlated with disease control and median progression-free survival in metastatic colorectal cancer patients treated with cetuximab or panitumumab (Yoshida et al. 2013). Similar to TGFα, there were consistent reports that found no significant correlation between HB-EGF mRNA expression and clinical outcome parameters in colorectal cancer patients treated with cetuximab (Baker et al. 2011; Cushman et al. 2015; Khambata-Ford et al. 2007). Furthermore, results obtained in head and neck cancer cells suggest that HB-EGF is a putative resistance factor against cetuximab (Hatakeyama et al. 2010).

To our knowledge, only little data are currently available regarding the value of HB-EGF as a predictive marker for the cetuximab or trastuzumab response in gastric cancer. In our study, only HB-EGF, but not AREG or EGF, rescued sensitive gastric cancer cell lines from trastuzumab and cetuximab treatment. As mentioned above, enhanced pHER2 expression has been associated with trastuzumab sensitivity in breast cancer cells (Diermeier et al. 2005; Dokmanovic et al. 2014). We were able to detect a comparable effect in the trastuzumab-sensitive cell line GSU. HB-EGF, but not AREG and EGF, blocked this induction pHER2 (Y1248) expression by trastuzumab effectively. We conclude that in our cell-based system, exogenous HB-EGF is a potent resistance factor against trastuzumab and cetuximab.

A literature search revealed that for most ligands, no consistent picture regarding their relationship with the clinical and prognostic features of gastric cancer was present. However, regarding different HB-EGF forms, all publications reported an association with advanced disease (Chung et al. 2015; Murayama et al. 2002; Shimura et al. 2012; Yasumoto et al. 2011). Additionally, there is evidence that HB-EGF is involved in the resistance of gastric tumours to chemotherapy, as HB-EGF expression was reported to be up-regulated in cisplatin- and 5-FU-resistant tumours (Suganuma et al. 2003). These findings were strengthened by cell culture-based results revealing cisplatin, 5-FU and paclitaxel stimulate HB-EGF secretion of gastric cancer cells. In this study, a synergistic antitumour effect was found when adding a HB-EGF inhibitor to paclitaxel (Sanui et al. 2010).

Based on work studying HB-EGF expression in advanced gastric cancer tumours, it is likely that HB-EGF contributes to the resistance of gastric tumours to trastuzumab- and cetuximab-based therapeutic approaches. The HB-EGF inhibitor CRM197 has been shown to be an effective inhibitor in gastric cancer cell lines, and further research in this field seems promising (Sanui et al. 2010). Moreover, the evaluation of HB-EGF as a prognostic marker in gastric cancer patients treated with trastuzumab-containing regimens should be considered.

## Electronic supplementary material

Below is the link to the electronic supplementary material.

Supplementary material 1 (PDF 1017 kb)

Supplementary material 2 (PDF 261 kb)

## Abbreviations

5-FU: 5-Fluorouracil
aCGH: Array-comparative genomic hybridization
AREG: Amphiregulin
CNV: Copy number variations
CRC: Colorectal cancer
DMEM: Dulbecco’s modified Eagle’s medium
ECACC: European Collection of Cell Cultures
EGF: Epidermal growth factor
EGFR: Epidermal growth factor receptor
ELISA: Enzyme-linked immunosorbent assay
EREG: Epiregulin
GEJ: Gastroesophageal junction
HB-EGF: Heparin-binding epidermal growth factor
HER: Human epidermal growth factor receptor
IgG: Immunoglobulin G
IU: International units
KRAS: Kirsten Ras gene
MEM: Minimum essential medium Eagle
NARD: Normalized amplicon read depth value
PBS: Phosphate-buffered saline
PCR: Polymerase chain reaction
PIK3CA: Phosphatidylinositol-4,5-bisphosphate 3-kinase, catalytic subunit alpha gene
PTEN: Phosphatase and tensin homologue
rpm: Rounds per minute
RTK: Receptor tyrosine kinase
SCCHN: Squamous cell carcinoma of the head and neck
SD: Standard deviation
TGFα: Transforming growth factor alpha

## References

[CR1] Aoyagi K, Kohfuji K, Yano S, Murakami N, Miyagi M, Takeda J, Shirouzu K (2001). Evaluation of the epidermal growth factor receptor (EGFR) and c-erbB-2 in superspreading-type and penetrating-type gastric carcinoma. Kurume Med J.

[CR2] Argiris A, Kotsakis AP, Hoang T, Worden FP, Savvides P, Gibson MK, Gyanchandani R, Blumenschein GR, Chen HX, Grandis JR (2013). Cetuximab and bevacizumab: preclinical data and phase II trial in recurrent or metastatic squamous cell carcinoma of the head and neck. Ann Oncol.

[CR3] Baker JB, Dutta D, Watson D, Maddala T, Munneke BM, Shak S, Rowinsky EK, Xu LA, Harbison CT, Clark EA (2011). Tumour gene expression predicts response to cetuximab in patients with KRAS wild-type metastatic colorectal cancer. Br J Cancer.

[CR4] Bang YJ, Van Cutsem E, Feyereislova A, Chung HC, Shen L, Sawaki A, Lordick F, Ohtsu A, Omuro Y, Satoh T (2010). Trastuzumab in combination with chemotherapy versus chemotherapy alone for treatment of HER2-positive advanced gastric or gastro-oesophageal junction cancer (ToGA): a phase 3, open-label, randomised controlled trial. Lancet.

[CR5] Beauchamp RD, Barnard JA, McCutchen CM, Cherner JA, Coffey RJ (1989). Localization of transforming growth factor alpha and its receptor in gastric mucosal cells. Implications for a regulatory role in acid secretion and mucosal renewal. J Clin Invest.

[CR6] Bennett C, Paterson IM, Corbishley CM, Luqmani YA (1989). Expression of growth factor and epidermal growth factor receptor encoded transcripts in human gastric tissues. Cancer Res.

[CR7] Berns K, Horlings HM, Hennessy BT, Madiredjo M, Hijmans EM, Beelen K, Linn SC, Gonzalez-Angulo AM, Stemke-Hale K, Hauptmann M (2007). A functional genetic approach identifies the PI3K pathway as a major determinant of trastuzumab resistance in breast cancer. Cancer Cell.

[CR8] Borlinghaus P, Wieser S, Lamerz R (1993). Epidermal growth factor, transforming growth factor-alpha, and epidermal growth factor receptor content in normal and carcinomatous gastric and colonic tissue. Clin Investig.

[CR9] Bremm A, Walch A, Fuchs M, Mages J, Duyster J, Keller G, Hermannstadter C, Becker KF, Rauser S, Langer R (2008). Enhanced activation of epidermal growth factor receptor caused by tumor-derived E-cadherin mutations. Cancer Res.

[CR10] Celikel C, Eren F, Gulluoglu B, Bekiroglu N, Turhal S (2007). Relation of neuroendocrine cells to transforming growth factor-alpha and epidermal growth factor receptor expression in gastric adenocarcinomas: prognostic implications. Pathol Oncol Res.

[CR11] Choi JH, Kim HC, Lim HY, Nam DK, Kim HS, Yi SY, Shim KS, Han WS (1999). Detection of transforming growth factor-alpha in the serum of gastric carcinoma patients. Oncology.

[CR12] Chuang LY, Hung WC, Yang ML, Chang CC, Tsai JF (1994). Urinary epidermal growth factor receptor-binding growth factors in patients with cancers of the digestive tract. Clin Biochem.

[CR13] Chung HW, Kong HY, Lim JB (2015). Clinical significance and usefulness of soluble heparin binding-epidermal growth factor in gastric cancer. World J Gastroenterol.

[CR14] Cushman SM, Jiang C, Hatch AJ, Shterev I, Sibley AB, Niedzwiecki D, Venook AP, Owzar K, Hurwitz HI, Nixon AB (2015). Gene expression markers of efficacy and resistance to cetuximab treatment in metastatic colorectal cancer: results from CALGB 80203 (Alliance). Clin Cancer Res.

[CR15] Czyzewska J, Guzinska-Ustymowicz K, Kemona A (2009). Correlation of c-erbB-2, EGF and EGFR expression with postoperative survival of patients with advanced carcinoma of the stomach. Folia Histochem Cytobiol/Pol Histochem Cytochem Soc.

[CR16] Debucquoy A, Haustermans K, Daemen A, Aydin S, Libbrecht L, Gevaert O, De Moor B, Tejpar S, McBride WH, Penninckx F (2009). Molecular response to cetuximab and efficacy of preoperative cetuximab-based chemoradiation in rectal cancer. J Clin Oncol.

[CR17] Dias A, Garcia C, Majewski M, Wallner G, McCallum RW, Poplawski C, Sarosiek J (2011). Gastric juice prostaglandins and peptide growth factors as potential markers of chronic atrophic gastritis, intestinal metaplasia and gastric cancer: their potential clinical implications based on this pilot study. Dig Dis Sci.

[CR18] Diermeier S, Horvath G, Knuechel-Clarke R, Hofstaedter F, Szollosi J, Brockhoff G (2005). Epidermal growth factor receptor coexpression modulates susceptibility to Herceptin in HER2/neu overexpressing breast cancer cells via specific erbB-receptor interaction and activation. Exp Cell Res.

[CR19] Docea AO, Mitrut P, Cernea D, Georgescu CC, Olimid D, Margaritescu C, Dumitrescu D (2013). Immunohistochemical expression of EGF, c-erbB-2 and EGFR in intestinal variant of gastric adenocarcinomas. Roman J Morphol Embryol/Rev Roum Morphol Embryol.

[CR20] Dokmanovic M, Wu Y, Shen Y, Chen J, Hirsch DS, Wu WJ (2014). Trastuzumab-induced recruitment of Csk-homologous kinase (CHK) to ErbB2 receptor is associated with ErbB2-Y1248 phosphorylation and ErbB2 degradation to mediate cell growth inhibition. Cancer Biol Ther.

[CR21] Dragovich T, McCoy S, Fenoglio-Preiser CM, Wang J, Benedetti JK, Baker AF, Hackett CB, Urba SG, Zaner KS, Blanke CD (2006). Phase II trial of erlotinib in gastroesophageal junction and gastric adenocarcinomas: SWOG 0127. J Clin Oncol.

[CR22] Endris V, Penzel R, Warth A, Muckenhuber A, Schirmacher P, Stenzinger A, Weichert W (2013). Molecular diagnostic profiling of lung cancer specimens with a semiconductor-based massive parallel sequencing approach: feasibility, costs, and performance compared with conventional sequencing. J Mol Diagn.

[CR23] Espinoza LA, Tone LG, Neto JB, Costa RS, Wang QJ, Ballejo G (2004). Enhanced TGFalpha-EGFR expression and P53 gene alterations contributes to gastric tumors aggressiveness. Cancer Lett.

[CR24] Fanelli MF, Chinen LT, Begnami MD, Costa WL, Fregnami JH, Soares FA, Montagnini AL (2012). The influence of transforming growth factor-alpha, cyclooxygenase-2, matrix metalloproteinase (MMP)-7, MMP-9 and CXCR4 proteins involved in epithelial-mesenchymal transition on overall survival of patients with gastric cancer. Histopathology.

[CR25] Ferlay J, Soerjomataram I, Dikshit R, Eser S, Mathers C, Rebelo M, Parkin DM, Forman D, Bray F (2015). Cancer incidence and mortality worldwide: sources, methods and major patterns in GLOBOCAN 2012. Int J Cancer.

[CR26] Forbes SA, Beare D, Gunasekaran P, Leung K, Bindal N, Boutselakis H, Ding M, Bamford S, Cole C, Ward S (2015). COSMIC: exploring the world’s knowledge of somatic mutations in human cancer. Nucleic Acids Res.

[CR27] Fukushige S, Matsubara K, Yoshida M, Sasaki M, Suzuki T, Semba K, Toyoshima K, Yamamoto T (1986). Localization of a novel v-erbB-related gene, c-erbB-2, on human chromosome 17 and its amplification in a gastric cancer cell line. Mol Cell Biol.

[CR28] Han SW, Oh DY, Im SA, Park SR, Lee KW, Song HS, Lee NS, Lee KH, Choi IS, Lee MH (2009). Phase II study and biomarker analysis of cetuximab combined with modified FOLFOX6 in advanced gastric cancer. Br J Cancer.

[CR29] Hatakeyama H, Cheng H, Wirth P, Counsell A, Marcrom SR, Wood CB, Pohlmann PR, Gilbert J, Murphy B, Yarbrough WG (2010). Regulation of heparin-binding EGF-like growth factor by miR-212 and acquired cetuximab-resistance in head and neck squamous cell carcinoma. PLoS ONE.

[CR30] Hecht JR, Bang YJ, Qin SK, Chung HC, Xu JM, Park JO, Jeziorski K, Shparyk Y, Hoff PM, Sobrero A (2016). Lapatinib in combination with capecitabine plus oxaliplatin in human epidermal growth factor receptor 2-positive advanced or metastatic gastric, esophageal, or gastroesophageal adenocarcinoma: TRIO-013/LOGiC—a randomized phase III trial. J Clin Oncol.

[CR31] Heindl S, Eggenstein E, Keller S, Kneissl J, Keller G, Mutze K, Rauser S, Gasteiger G, Drexler I, Hapfelmeier A (2012). Relevance of MET activation and genetic alterations of KRAS and E-cadherin for cetuximab sensitivity of gastric cancer cell lines. J Cancer Res Clin Oncol.

[CR32] Hinoda Y, Sasaki S, Ishida T, Imai K (2004). Monoclonal antibodies as effective therapeutic agents for solid tumors. Cancer Sci.

[CR33] Hirata Y, Ogasawara N, Sasaki M, Mizushima T, Shimura T, Mizoshita T, Mori Y, Kubota E, Wada T, Tanida S (2009). BCL6 degradation caused by the interaction with the C-terminus of pro-HB-EGF induces cyclin D2 expression in gastric cancers. Br J Cancer.

[CR34] Hirayama D, Fujimori T, Satonaka K, Nakamura T, Kitazawa S, Horio M, Maeda S, Nagasako K (1992). Immunohistochemical study of epidermal growth factor and transforming growth factor-beta in the penetrating type of early gastric cancer. Hum Pathol.

[CR35] Hobor S, Van Emburgh BO, Crowley E, Misale S, Di Nicolantonio F, Bardelli A (2014). TGFalpha and amphiregulin paracrine network promotes resistance to EGFR blockade in colorectal cancer cells. Clin Cancer Res.

[CR36] Jacobs B, De Roock W, Piessevaux H, Van Oirbeek R, Biesmans B, De Schutter J, Fieuws S, Vandesompele J, Peeters M, Van Laethem JL (2009). Amphiregulin and epiregulin mRNA expression in primary tumors predicts outcome in metastatic colorectal cancer treated with cetuximab. J Clin Oncol.

[CR37] Jonker DJ, Karapetis CS, Harbison C, O’Callaghan CJ, Tu D, Simes RJ, Malone DP, Langer C, Tebbutt N, Price TJ (2014). Epiregulin gene expression as a biomarker of benefit from cetuximab in the treatment of advanced colorectal cancer. Br J Cancer.

[CR38] Juskevicius D, Lorber T, Gsponer J, Perrina V, Ruiz C, Stenner-Liewen F, Dirnhofer S, Tzankov A (2016). Distinct genetic evolution patterns of relapsing diffuse large B-cell lymphoma revealed by genome-wide copy number aberration and targeted sequencing analysis. Leukemia.

[CR39] Kang YK, Rha SY, Tassone P, Barriuso J, Yu R, Szado T, Garg A, Bang YJ (2014). A phase IIa dose-finding and safety study of first-line pertuzumab in combination with trastuzumab, capecitabine and cisplatin in patients with HER2-positive advanced gastric cancer. Br J Cancer.

[CR40] Karapetis CS, Khambata-Ford S, Jonker DJ, O’Callaghan CJ, Tu D, Tebbutt NC, Simes RJ, Chalchal H, Shapiro JD, Robitaille S (2008). K-ras mutations and benefit from cetuximab in advanced colorectal cancer. N Engl J Med.

[CR41] Khambata-Ford S, Garrett CR, Meropol NJ, Basik M, Harbison CT, Wu S, Wong TW, Huang X, Takimoto CH, Godwin AK (2007). Expression of epiregulin and amphiregulin and K-ras mutation status predict disease control in metastatic colorectal cancer patients treated with cetuximab. J Clin Oncol.

[CR42] Kim IJ, Park JH, Kang HC, Shin Y, Park HW, Park HR, Ku JL, Lim SB, Park JG (2003). Mutational analysis of BRAF and K-ras in gastric cancers: absence of BRAF mutations in gastric cancers. Hum Genet.

[CR43] Kim JY, Jeon TJ, Bae BN, Kwon JE, Kim HJ, Park K, Shin E (2013). The prognostic significance of growth factors and growth factor receptors in gastric adenocarcinoma. APMIS.

[CR44] Kim JW, Kim DK, Min A, Lee KH, Nam HJ, Kim JH, Kim JS, Kim TY, Im SA, Park IA (2015). Amphiregulin confers trastuzumab resistance via AKT and ERK activation in HER2-positive breast cancer. J Cancer Res Clin Oncol.

[CR45] Kitadai Y, Yasui W, Yokozaki H, Kuniyasu H, Ayhan A, Haruma K, Kajiyama G, Johnson GR, Tahara E (1993). Expression of amphiregulin, a novel gene of the epidermal growth factor family, in human gastric carcinomas. Jpn J Cancer Res.

[CR46] Kneissl J, Keller S, Lorber T, Heindl S, Keller G, Drexler I, Hapfelmeier A, Hofler H, Luber B (2012). Association of amphiregulin with the cetuximab sensitivity of gastric cancer cell lines. Int J Oncol.

[CR47] Konturek PC, Konturek SJ, Sulekova Z, Meixner H, Bielanski W, Starzynska T, Karczewska E, Marlicz K, Stachura J, Hahn EG (2001). Expression of hepatocyte growth factor, transforming growth factor alpha, apoptosis related proteins Bax and Bcl-2, and gastrin in human gastric cancer. Aliment Pharmacol Ther.

[CR48] Kubo T, Kuroda Y, Shimizu H, Kokubu A, Okada N, Hosoda F, Arai Y, Nakamura Y, Taniguchi H, Yanagihara K (2009). Resequencing and copy number analysis of the human tyrosine kinase gene family in poorly differentiated gastric cancer. Carcinogenesis.

[CR49] Kuramochi H, Nakajima G, Kaneko Y, Nakamura A, Inoue Y, Yamamoto M, Hayashi K (2012). Amphiregulin and Epiregulin mRNA expression in primary colorectal cancer and corresponding liver metastases. BMC Cancer.

[CR50] Leto SM, Sassi F, Catalano I, Torri V, Migliardi G, Zanella ER, Throsby M, Bertotti A, Trusolino L (2015). Sustained inhibition of HER3 and EGFR is necessary to induce regression of HER2-amplified gastrointestinal carcinomas. Clin Cancer Res.

[CR51] Li J, Davies BR, Han S, Zhou M, Bai Y, Zhang J, Xu Y, Tang L, Wang H, Liu YJ (2013). The AKT inhibitor AZD5363 is selectively active in PI3KCA mutant gastric cancer, and sensitizes a patient-derived gastric cancer xenograft model with PTEN loss to Taxotere. J Transl Med.

[CR52] Lievre A, Bachet JB, Le Corre D, Boige V, Landi B, Emile JF, Cote JF, Tomasic G, Penna C, Ducreux M (2006). KRAS mutation status is predictive of response to cetuximab therapy in colorectal cancer. Cancer Res.

[CR53] Liu Y, Ling Y, Qi Q, Zhu M, Wan M, Zhang Y, Zhang C (2015). Trastuzumab increases the sensitivity of HER2-amplified human gastric cancer cells to oxaliplatin and cisplatin by affecting the expression of telomere-associated proteins. Oncol Lett.

[CR54] Livingstone JI, Yasui W, Tahara E, Wastell C (1995). Are Japanese and European gastric cancer the same biological entity? An immunohistochemical study. Br J Cancer.

[CR55] Lordick F, Kang YK, Chung HC, Salman P, Oh SC, Bodoky G, Kurteva G, Volovat C, Moiseyenko VM, Gorbunova V (2013). Capecitabine and cisplatin with or without cetuximab for patients with previously untreated advanced gastric cancer (EXPAND): a randomised, open-label phase 3 trial. Lancet Oncol.

[CR56] Lorenzen S, Riera Knorrenschild J, Haag GM, Pohl M, Thuss-Patience P, Bassermann F, Helbig U, Weissinger F, Schnoy E, Becker K (2015). Lapatinib versus lapatinib plus capecitabine as second-line treatment in human epidermal growth factor receptor 2-amplified metastatic gastro-oesophageal cancer: a randomised phase II trial of the Arbeitsgemeinschaft Internistische Onkologie. Eur J Cancer.

[CR57] Loupakis F, Cremolini C, Fioravanti A, Orlandi P, Salvatore L, Masi G, Schirripa M, Di Desidero T, Antoniotti C, Canu B (2014). EGFR ligands as pharmacodynamic biomarkers in metastatic colorectal cancer patients treated with cetuximab and irinotecan. Target Oncol.

[CR58] Majewski IJ, Nuciforo P, Mittempergher L, Bosma AJ, Eidtmann H, Holmes E, Sotiriou C, Fumagalli D, Jimenez J, Aura C (2015). PIK3CA mutations are associated with decreased benefit to neoadjuvant human epidermal growth factor receptor 2-targeted therapies in breast cancer. J Clin Oncol.

[CR59] Mita H, Toyota M, Aoki F, Akashi H, Maruyama R, Sasaki Y, Suzuki H, Idogawa M, Kashima L, Yanagihara K (2009). A novel method, digital genome scanning detects KRAS gene amplification in gastric cancers: involvement of overexpressed wild-type KRAS in downstream signaling and cancer cell growth. BMC Cancer.

[CR60] Mochizuki S, Okada Y (2007). ADAMs in cancer cell proliferation and progression. Cancer Sci.

[CR61] Moskal TL, Huang S, Ellis LM, Fritsche HA, Chakrabarty S (1995). Serum levels of transforming growth factor alpha in gastrointestinal cancer patients. Cancer Epidemiol Biomark Prev.

[CR62] Muller W, Borchard F (1992). Expression of transforming growth factor-alpha in gastric carcinoma and normal gastric mucosa cells. Cancer.

[CR63] Murayama Y, Miyagawa J, Shinomura Y, Kanayama S, Isozaki K, Yamamori K, Mizuno H, Ishiguro S, Kiyohara T, Miyazaki Y (2002). Significance of the association between heparin-binding epidermal growth factor-like growth factor and CD9 in human gastric cancer. Int J Cancer.

[CR64] Mutsaers AJ, Francia G, Man S, Lee CR, Ebos JM, Wu Y, Witte L, Berry S, Moore M, Kerbel RS (2009). Dose-dependent increases in circulating TGF-alpha and other EGFR ligands act as pharmacodynamic markers for optimal biological dosing of cetuximab and are tumor independent. Clin Cancer Res.

[CR65] Naef M, Yokoyama M, Friess H, Buchler MW, Korc M (1996). Co-expression of heparin-binding EGF-like growth factor and related peptides in human gastric carcinoma. Int J Cancer.

[CR66] Nam HJ, Ching KA, Kan J, Kim HP, Han SW, Im SA, Kim TY, Christensen JG, Oh DY, Bang YJ (2012). Evaluation of the antitumor effects and mechanisms of PF00299804, a pan-HER inhibitor, alone or in combination with chemotherapy or targeted agents in gastric cancer. Mol Cancer Ther.

[CR67] Nasim MM, Thomas DM, Alison MR, Filipe MI (1992). Transforming growth factor alpha expression in normal gastric mucosa, intestinal metaplasia, dysplasia and gastric carcinoma—an immunohistochemical study. Histopathology.

[CR68] Nielsen TO, Friis-Hansen L, Poulsen SS, Federspiel B, Sorensen BS (2014). Expression of the EGF family in gastric cancer: downregulation of HER4 and its activating ligand NRG4. PLoS ONE.

[CR69] Oda N, Tsujino T, Tsuda T, Yoshida K, Nakayama H, Yasui W, Tahara E (1990). DNA ploidy pattern and amplification of ERBB and ERBB2 genes in human gastric carcinomas. Virchows Archiv B.

[CR70] Okines A, Verheij M, Allum W, Cunningham D, Cervantes A, Grou EGW (2010). Gastric cancer: ESMO Clinical Practice Guidelines for diagnosis, treatment and follow-up. Ann Oncol.

[CR71] Onda M, Tokunaga A, Nishi K, Yoshiyuki T, Shimizu Y, Kiyama T, Mizutani T, Matsukura N, Tanaka N, Yamashita K (1990). The correlation of epidermal growth factor with invasion and metastasis in human gastric cancer. Jpn J Surg.

[CR72] Park do J, Yoon C, Thomas N, Ku GY, Janjigian YY, Kelsen DP, Ilson DH, Goodman KA, Tang LH, Strong VE (2014). Prognostic significance of targetable angiogenic and growth factors in patients undergoing resection for gastric and gastroesophageal junction cancers. Ann Surg Oncol.

[CR73] Pentheroudakis G, Kotoula V, De Roock W, Kouvatseas G, Papakostas P, Makatsoris T, Papamichael D, Xanthakis I, Sgouros J, Televantou D (2013). Biomarkers of benefit from cetuximab-based therapy in metastatic colorectal cancer: interaction of EGFR ligand expression with RAS/RAF, PIK3CA genotypes. BMC Cancer.

[CR74] Pryczynicz A, Guzinska-Ustymowicz K, Kemona A, Czyzewska J (2009). Helicobacter pylori infection and expressions of EGF, EGFR and c-erbB-2 proteins in gastric carcinoma. Folio Histochem Cytobiol/Pol Histochem Cytochem Soc.

[CR75] Rajcevic U, Juvan R, Gazvoda B, Repse S, Komel R (2001). Assessment of differential expression of oncogenes in gastric adenocarcinoma by fluorescent multiplex RT-PCR assay. Pflugers Arch.

[CR76] Rhee J, Han SW, Cha Y, Ham HS, Kim HP, Oh DY, Im SA, Park JW, Ro J, Lee KS (2011). High serum TGF-alpha predicts poor response to lapatinib and capecitabine in HER2-positive breast cancer. Breast Cancer Res Treat.

[CR77] Ritter CA, Perez-Torres M, Rinehart C, Guix M, Dugger T, Engelman JA, Arteaga CL (2007). Human breast cancer cells selected for resistance to trastuzumab in vivo overexpress epidermal growth factor receptor and ErbB ligands and remain dependent on the ErbB receptor network. Clin Cancer Res.

[CR78] Ruiz C, Lenkiewicz E, Evers L, Holley T, Robeson A, Kiefer J, Demeure MJ, Hollingsworth MA, Shen M, Prunkard D (2011). Advancing a clinically relevant perspective of the clonal nature of cancer. Proc Natl Acad Sci USA.

[CR79] Saeki T, Salomon D, Normanno N, Johnson G, Gullick W, Mandai K, Moriwaki S, Takashima S, Kuniyasu M, Tahara E (1994). Immunohistochemical detection of cripto-1, amphiregulin and transforming growth-factor-alpha in human gastric carcinomas and intestinal metaplasias. Int J Oncol.

[CR80] Saki M, Toulany M, Rodemann HP (2013). Acquired resistance to cetuximab is associated with the overexpression of Ras family members and the loss of radiosensitization in head and neck cancer cells. Radiother Oncol.

[CR81] Sanui A, Yotsumoto F, Tsujioka H, Fukami T, Horiuchi S, Shirota K, Yoshizato T, Kawarabayashi T, Kuroki M, Miyamoto S (2010). HB-EGF inhibition in combination with various anticancer agents enhances its antitumor effects in gastric cancer. Anticancer Res.

[CR82] Satoh T, Xu RH, Chung HC, Sun GP, Doi T, Xu JM, Tsuji A, Omuro Y, Li J, Wang JW (2014). Lapatinib plus paclitaxel versus paclitaxel alone in the second-line treatment of HER2-amplified advanced gastric cancer in Asian populations: TyTAN—a randomized, phase III study. J Clin Oncol.

[CR83] Sherry ST, Ward MH, Kholodov M, Baker J, Phan L, Smigielski EM, Sirotkin K (2001). dbSNP: the NCBI database of genetic variation. Nucleic Acids Res.

[CR84] Shimoyama S (2014). Unraveling trastuzumab and lapatinib inefficiency in gastric cancer: future steps (review). Mol Clin Oncol.

[CR85] Shimura T, Yoshida M, Fukuda S, Ebi M, Hirata Y, Mizoshita T, Tanida S, Kataoka H, Kamiya T, Higashiyama S (2012). Nuclear translocation of the cytoplasmic domain of HB-EGF induces gastric cancer invasion. BMC Cancer.

[CR86] Stenzinger A, Endris V, Pfarr N, Andrulis M, Johrens K, Klauschen F, Siebolts U, Wolf T, Koch PS, Schulz M (2014). Targeted ultra-deep sequencing reveals recurrent and mutually exclusive mutations of cancer genes in blastic plasmacytoid dendritic cell neoplasm. Oncotarget.

[CR87] Sternlicht MD, Sunnarborg SW (2008). The ADAM17-amphiregulin-EGFR axis in mammary development and cancer. J Mammary Gland Biol Neoplasia.

[CR88] Suganuma K, Kubota T, Saikawa Y, Abe S, Otani Y, Furukawa T, Kumai K, Hasegawa H, Watanabe M, Kitajima M (2003). Possible chemoresistance-related genes for gastric cancer detected by cDNA microarray. Cancer Sci.

[CR89] Sugiyama K, Yonemura Y, Miyazaki I (1989). Immunohistochemical study of epidermal growth factor and epidermal growth factor receptor in gastric carcinoma. Cancer.

[CR90] Tahara E, Sumiyoshi H, Hata J, Yasui W, Taniyama K, Hayashi T, Nagae S, Sakamoto S (1986). Human epidermal growth factor in gastric carcinoma as a biologic marker of high malignancy. Jpn J Cancer Res.

[CR91] Takada H, Imoto I, Tsuda H, Sonoda I, Ichikura T, Mochizuki H, Okanoue T, Inazawa J (2005). Screening of DNA copy-number aberrations in gastric cancer cell lines by array-based comparative genomic hybridization. Cancer Sci.

[CR92] Takahashi N, Yamada Y, Furuta K, Honma Y, Iwasa S, Takashima A, Kato K, Hamaguchi T, Shimada Y (2014). Serum levels of hepatocyte growth factor and epiregulin are associated with the prognosis on anti-EGFR antibody treatment in KRAS wild-type metastatic colorectal cancer. Br J Cancer.

[CR93] Takita M, Onda M, Tokunaga A (1998). Immunohistochemical demonstration of angiogenic growth factors and EGF receptor in hepatic metastases and primary human gastric cancer. Nihon Ika Daigaku zasshi.

[CR94] Tomioka H, Mukohara T, Kataoka Y, Ekyalongo RC, Funakoshi Y, Imai Y, Kiyota N, Fujiwara Y, Minami H (2012). Inhibition of the mTOR/S6K signal is necessary to enhance fluorouracil-induced apoptosis in gastric cancer cells with HER2 amplification. Int J Oncol.

[CR95] Troiani T, Martinelli E, Napolitano S, Vitagliano D, Ciuffreda LP, Costantino S, Morgillo F, Capasso A, Sforza V, Nappi A (2013). Increased TGF-alpha as a mechanism of acquired resistance to the anti-EGFR inhibitor cetuximab through EGFR-MET interaction and activation of MET signaling in colon cancer cells. Clin Cancer Res.

[CR96] Valabrega G, Montemurro F, Sarotto I, Petrelli A, Rubini P, Tacchetti C, Aglietta M, Comoglio PM, Giordano S (2005). TGFalpha expression impairs trastuzumab-induced HER2 downregulation. Oncogene.

[CR97] Waddell T, Chau I, Cunningham D, Gonzalez D, Okines AF, Okines C, Wotherspoon A, Saffery C, Middleton G, Wadsley J (2013). Epirubicin, oxaliplatin, and capecitabine with or without panitumumab for patients with previously untreated advanced oesophagogastric cancer (REAL3): a randomised, open-label phase 3 trial. Lancet Oncol.

[CR98] Wainberg ZA, Anghel A, Desai AJ, Ayala R, Luo T, Safran B, Fejzo MS, Hecht JR, Slamon DJ, Finn RS (2010). Lapatinib, a dual EGFR and HER2 kinase inhibitor, selectively inhibits HER2-amplified human gastric cancer cells and is synergistic with trastuzumab in vitro and in vivo. Clin Cancer Res.

[CR99] Wilgenbus K, Lentner A, Kuckelkorn R, Handt S, Mittermayer C (1992). Further evidence that acanthosis nigricans maligna is linked to enhanced secretion by the tumour of transforming growth factor alpha. Arch Dermatol Res.

[CR100] Yamashita-Kashima Y, Iijima S, Yorozu K, Furugaki K, Kurasawa M, Ohta M, Fujimoto-Ouchi K (2011). Pertuzumab in combination with trastuzumab shows significantly enhanced antitumor activity in HER2-positive human gastric cancer xenograft models. Clin Cancer Res.

[CR101] Yasui W, Hata J, Yokozaki H, Nakatani H, Ochiai A, Ito H, Tahara E (1988). Interaction between epidermal growth factor and its receptor in progression of human gastric carcinoma. Int J Cancer.

[CR102] Yasumoto K, Yamada T, Kawashima A, Wang W, Li Q, Donev IS, Tacheuchi S, Mouri H, Yamashita K, Ohtsubo K (2011). The EGFR ligands amphiregulin and heparin-binding egf-like growth factor promote peritoneal carcinomatosis in CXCR4-expressing gastric cancer. Clin Cancer Res.

[CR103] Yoshida K, Kyo E, Tsujino T, Sano T, Niimoto M, Tahara E (1990). Expression of epidermal growth factor, transforming growth factor-alpha and their receptor genes in human gastric carcinomas; implication for autocrine growth. Jpn J Cancer Res.

[CR104] Yoshida M, Shimura T, Fukuda S, Mizoshita T, Tanida S, Kataoka H, Kamiya T, Nakazawa T, Higashiyama S, Joh T (2012). Nuclear translocation of pro-amphiregulin induces chemoresistance in gastric cancer. Cancer Sci.

[CR105] Yoshida M, Shimura T, Sato M, Ebi M, Nakazawa T, Takeyama H, Joh T (2013). A novel predictive strategy by immunohistochemical analysis of four EGFR ligands in metastatic colorectal cancer treated with anti-EGFR antibodies. J Cancer Res Clin Oncol.

[CR106] Yoshiyuki T, Shimizu Y, Onda M, Tokunaga A, Kiyama T, Nishi K, Mizutani T, Matsukura N, Tanaka N, Akimoto M (1990). Immunohistochemical demonstration of epidermal growth factor in human gastric cancer xenografts of nude mice. Cancer.

[CR107] Yotsumoto F, Oki E, Tokunaga E, Maehara Y, Kuroki M, Miyamoto S (2010). HB-EGF orchestrates the complex signals involved in triple-negative and trastuzumab-resistant breast cancer. Int J Cancer.

[CR108] Zang ZJ, Ong CK, Cutcutache I, Yu W, Zhang SL, Huang D, Ler LD, Dykema K, Gan A, Tao J (2011). Genetic and structural variation in the gastric cancer kinome revealed through targeted deep sequencing. Cancer Res.

[CR109] Zhang X, Xu J, Liu H, Yang L, Liang J, Xu N, Bai Y, Wang J, Shen L (2014). Predictive biomarkers for the efficacy of cetuximab combined with cisplatin and capecitabine in advanced gastric or esophagogastric junction adenocarcinoma: a prospective multicenter phase 2 trial. Med Oncol.

[CR110] Zhou XK, Tang SS, Yi G, Hou M, Chen JH, Yang B, Liu JF, He ZM (2011). RNAi knockdown of PIK3CA preferentially inhibits invasion of mutant PIK3CA cells. World J Gastroenterol.

