# Supplementary material for: Influence of the HER receptor ligand system on sensitivity to cetuximab and trastuzumab in gastric cancer cell lines
Source: J Cancer Res Clin Oncol. 2016 Dec 8;143(4):573–600. doi: 10.1007/s00432-016-2308-z (PMC5352771; doi:10.1007/s00432-016-2308-z)
Supplement: Supplementary file 1 — Supplementary material 1 (PDF 1017 kb) [file 432_2016_2308_MOESM1_ESM.pdf]

Influence of the Her-receptor ligand system on sensitivity to cetuximab and trastuzumab in gastric cancer cell lines

Journal of Cancer Research and Clinical Oncology

Julia Kneissl, Anja Hartmann, Nicole Pfarr, Franziska Erlmeier, Thomas Lorber, Simone Keller, Gwen Zwingenberger, Wilko Weichert, Birgit Luber

Correspondence to: Dr. Birgit Luber, Institute of Pathology, Technische Universität München, Klinikum rechts der Isar, Trogerstr. 18, D-81675 München, Germany, E-mail: birgit.luber@tum.de

Online Resource 1:

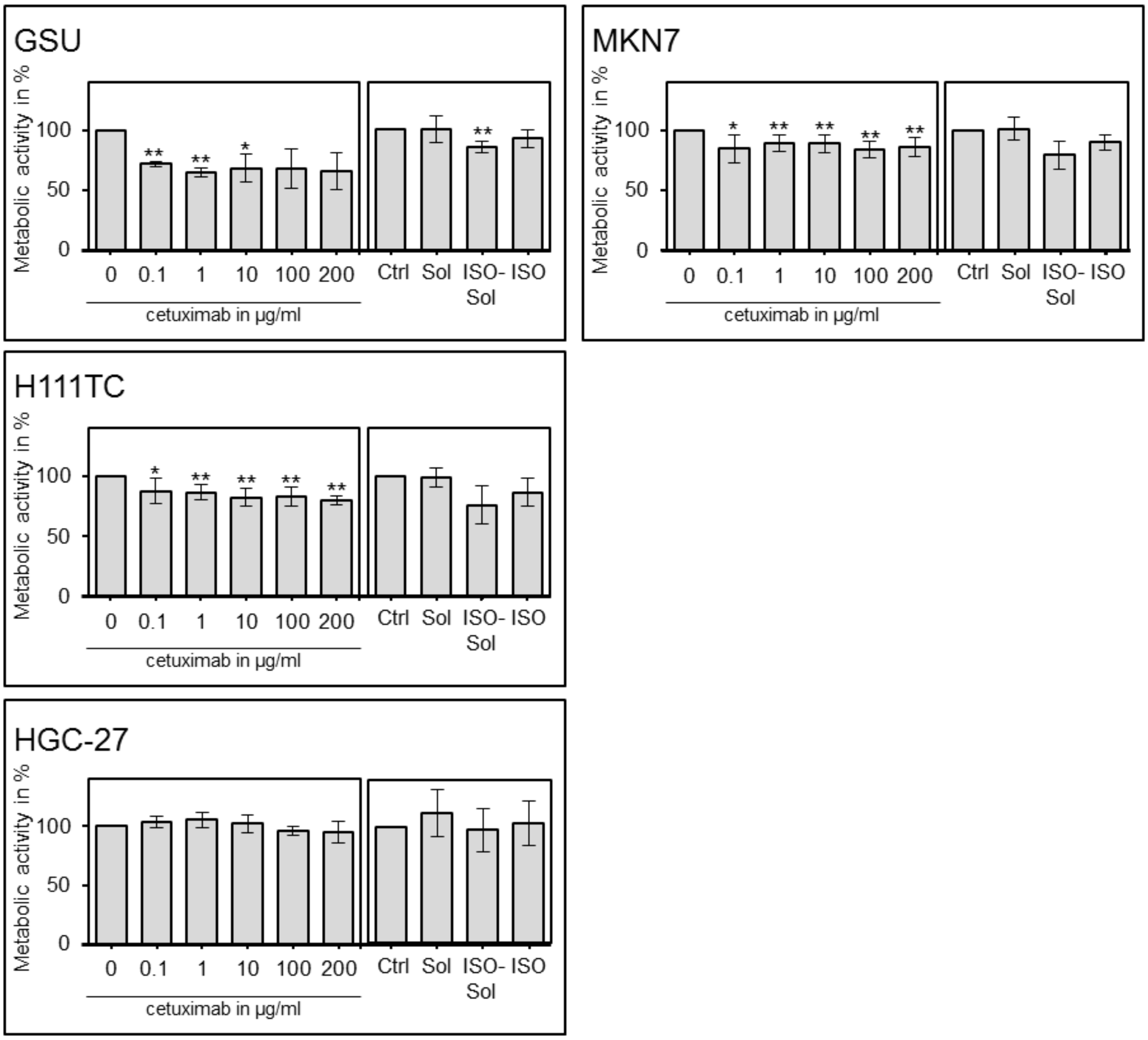

Online Resource 2:
Suspected copy number variations in the gastric cancer cell lines GSU and H111TC

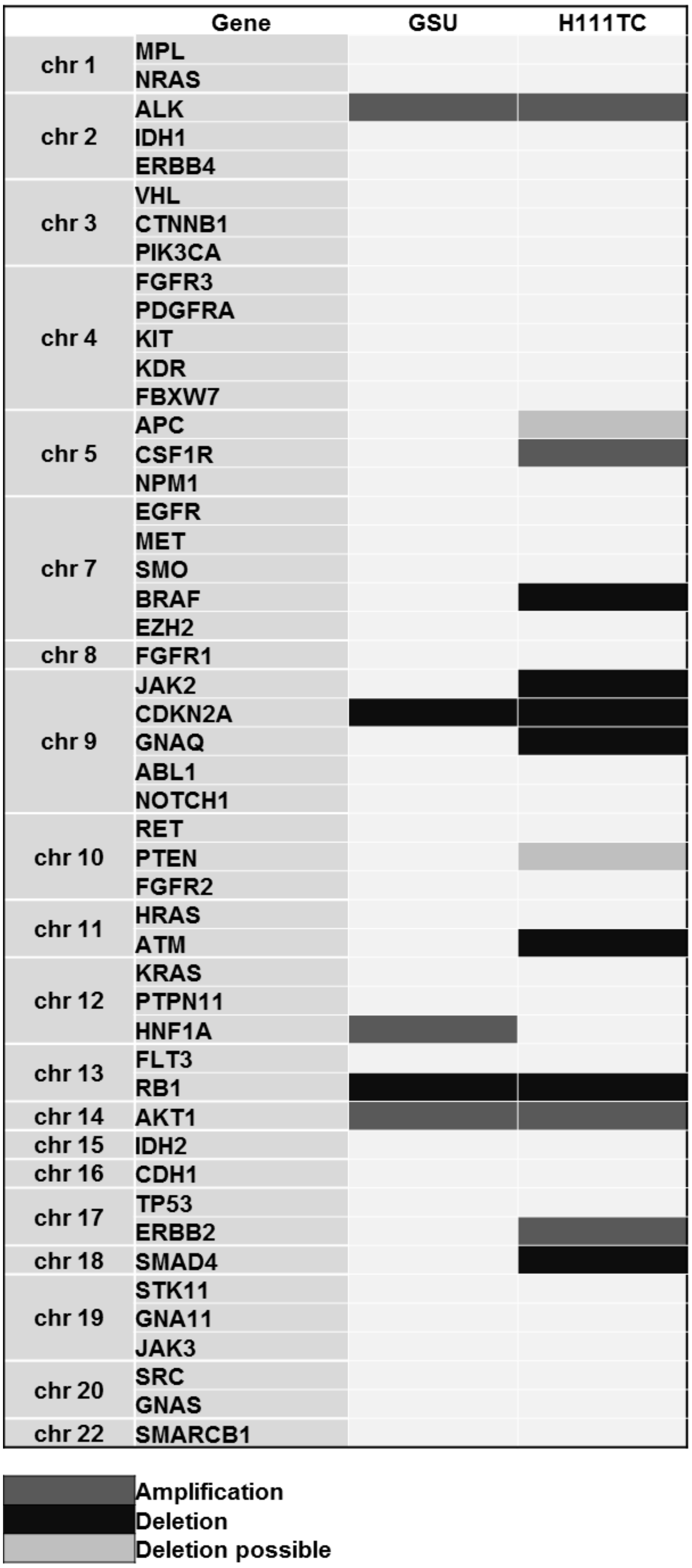

Online Resource 3:

Array-comparative genomic hybridization analysis for copy number status of locus *ERBB2* (*HER2*) for cell lines GSU (a) and H111TC (b).

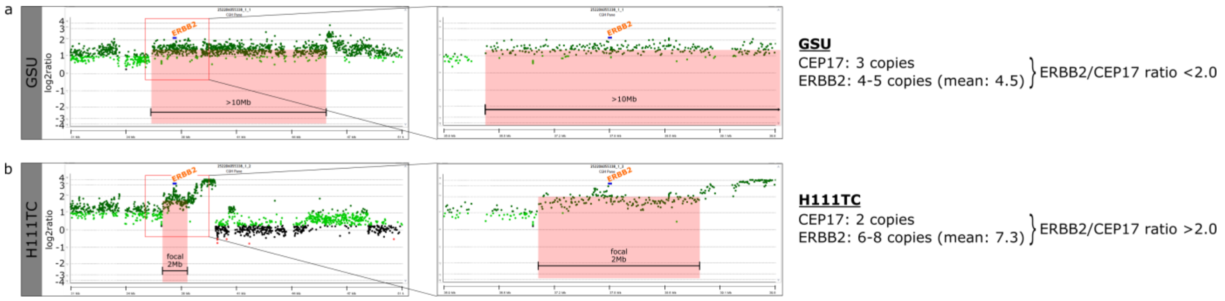

Online Resource 4:
Amplifications and deletions of selected genes in GSU and H111TC cells

		GSU	H111TC
	mean CN of cell line	3.9	2.6
	**Gene**	CN	
**chr 1**	**MPL**	3	4
	**NRAS**	3	2
**chr 2**	**ALK**	4	4
	**IDH1**	4	4
	**ERBB4**	4	4
**chr 3**	**VHL**	5	2
	**CTNNB1**	3	2
	**PIK3CA**	3	8
**chr 4**	**FGFR3**	3	2
	**PDGFRA**	3	3
	**KIT**	3	3
	**KDR**	3	3
	**FBXW7**	3	2
**chr 5**	**APC**	3	2
	**CSF1R**	3	4
	**NPM1**	3	4
**chr 7**	**EGFR**	4	3
	**MET**	4	4
	**SMO**	4	2
	**BRAF**	4	2
	**EZH2**	4	2
**chr 8**	**FGFR1**	4	7
**chr 9**	**JAK2**	3	1
	**CDKN2A**	1	1
	**GNAQ**	3	2
	**ABL1**	3	3
	**NOTCH1**	3	3
**chr 10**	**RET**	3	2
	**PTEN**	4	2
	**FGFR2**	4	3
**chr 11**	**HRAS**	4	5
	**ATM**	4	1
**chr 12**	**KRAS**	4	3
	**PTPN11**	4	3
	**HNF1A**	>10	4
**chr 13**	**FLT3**	3	3
	**RB1**	3	3
**chr 14**	**AKT1**	4	3
**chr 15**	**IDH2**	4	5
**chr 16**	**CDH1**	4	4
**chr 17**	**TP53**	4	2
	**ERBB2**	4-5	6-8
**chr 18**	**SMAD4**	3	1
**chr 19**	**STK11**	4	2
	**GNA11**	3	2
	**JAK3**	3	2
**chr 20**	**SRC**	3	4
	**GNAS**	3	4
**chr 22**	**SMARCB1**	3	4

	CN of gene / mean CN of cell line
Deletion	<0.5
Amplification	>2

CN: copy number

Online Resource 5:

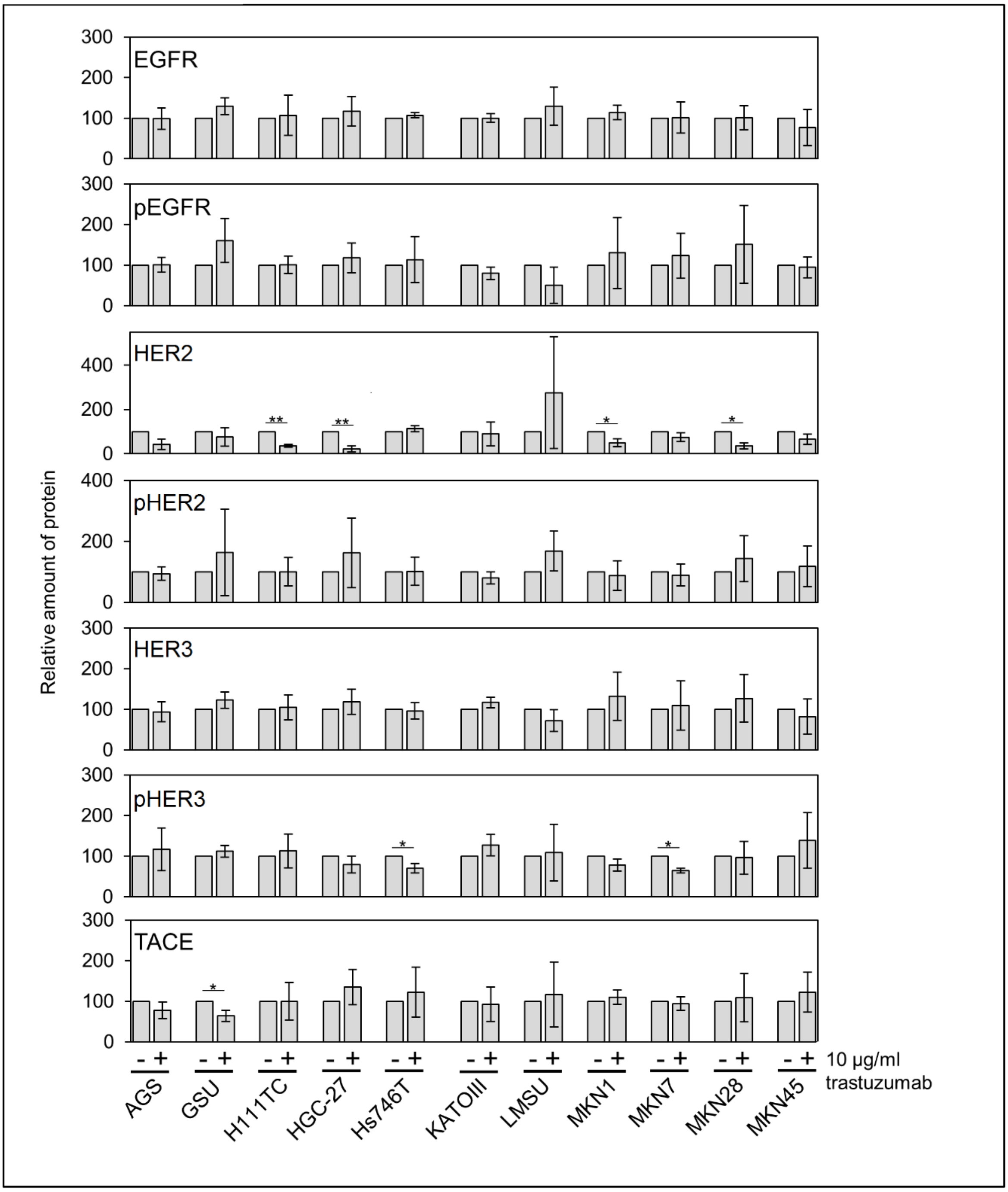

Online Resource 6:

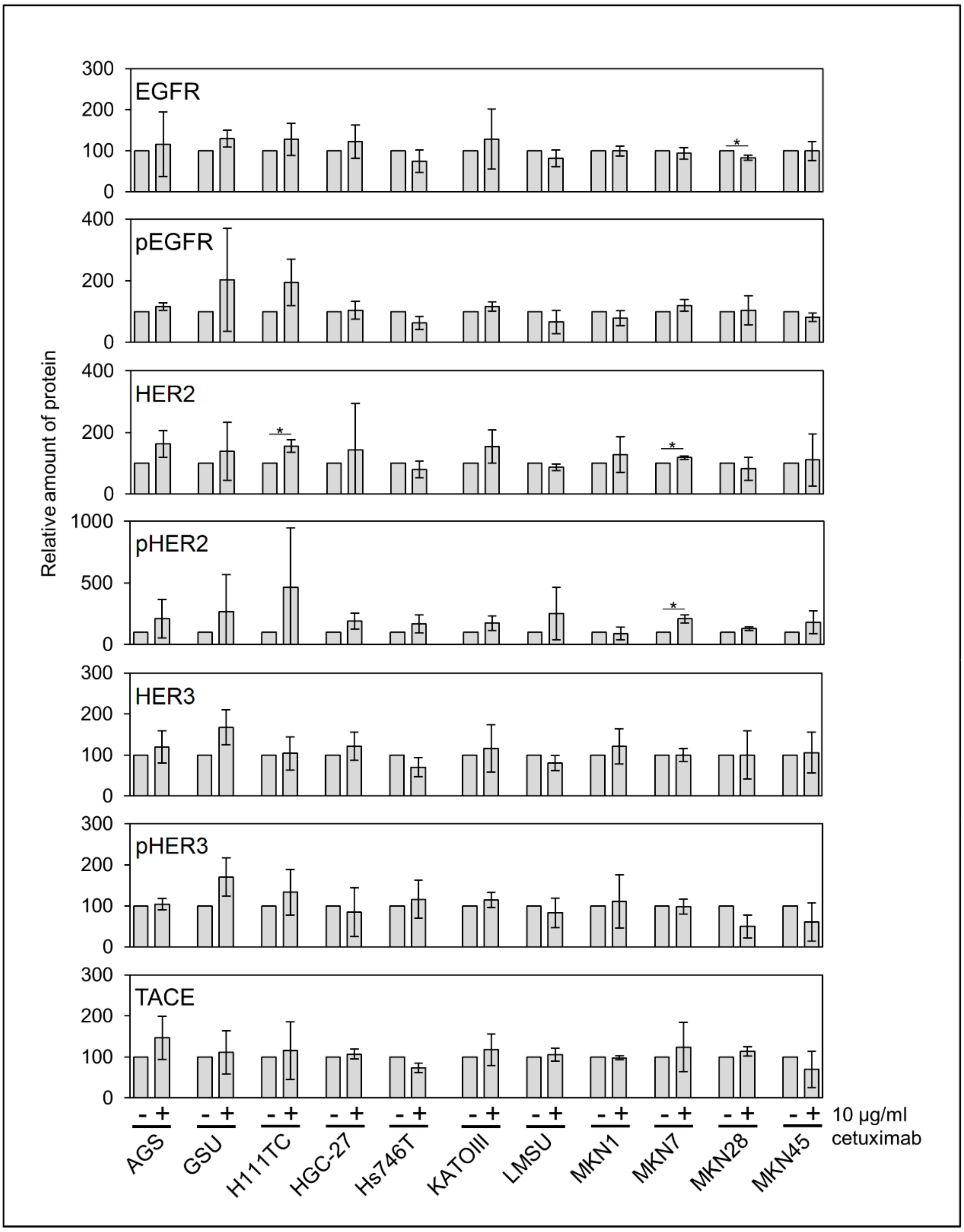

Online Resource 7:

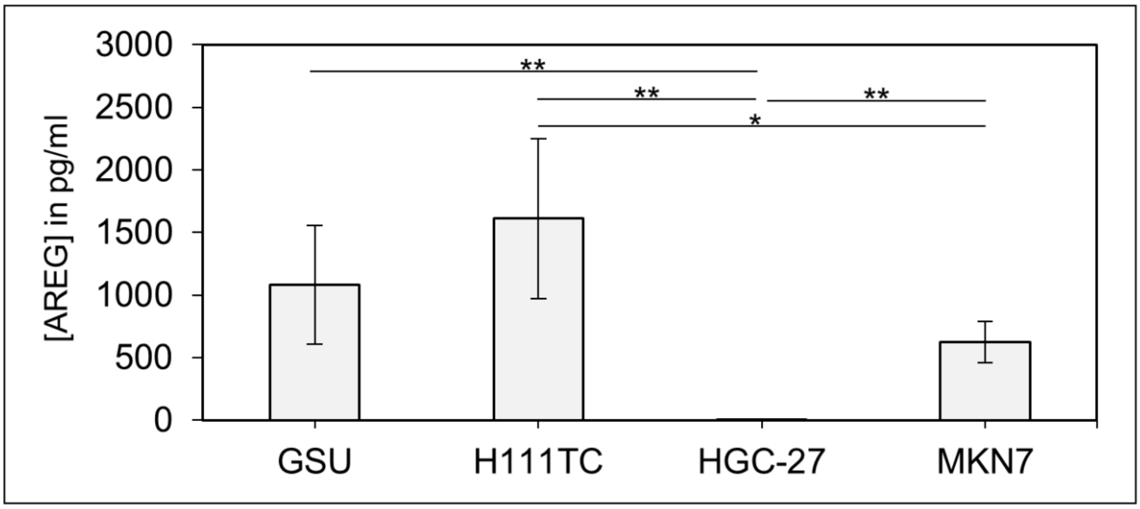

Online Resource 9

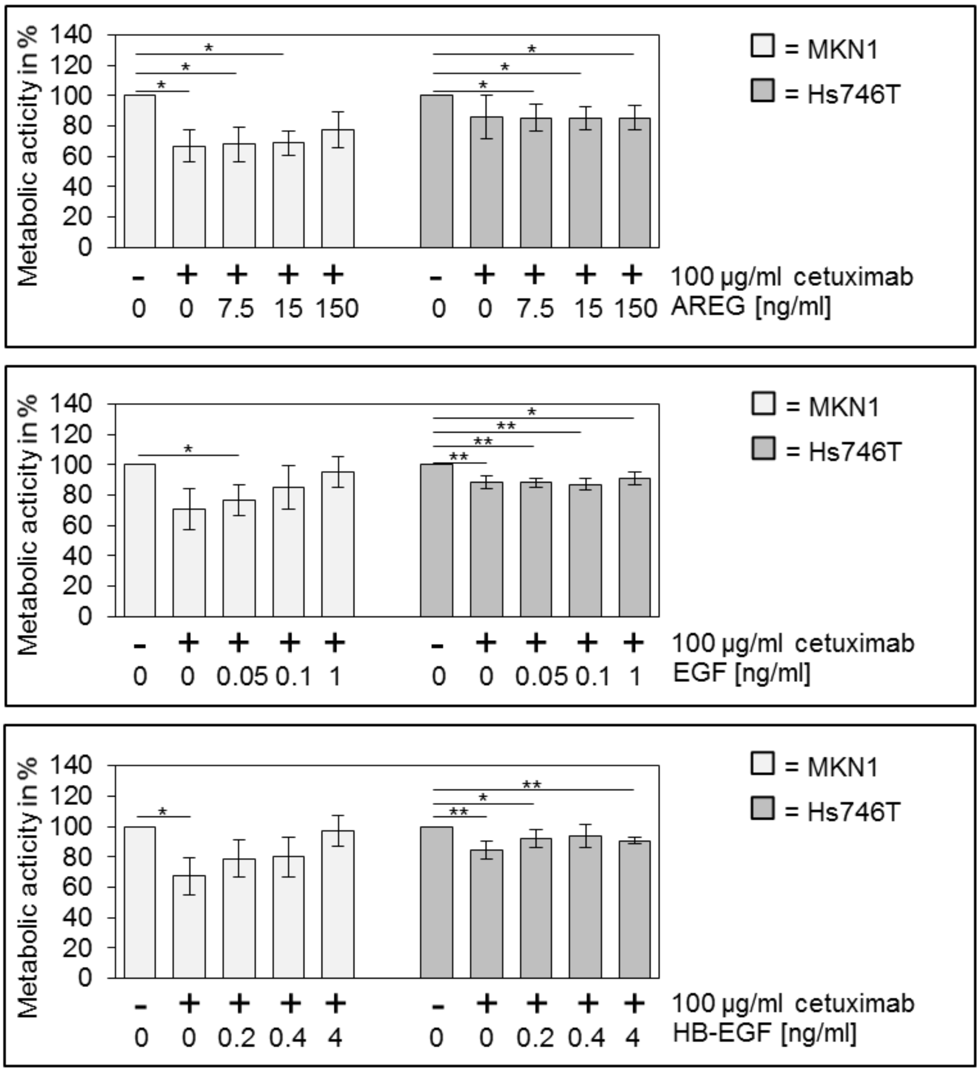

Figure legends

Online Resource 1 Effect of cetuximab treatment on the metabolic activity of the gastric cancer cell lines GSU, H111TC, HGC-27, and MKN7

The cell lines were treated for 48 h with the indicated amounts of cetuximab (0 / 0.1 / 1 / 10 / 100 / 200 µg/ml), a solvent control (Sol), an isotype control (ISO) or isotype solvent control (ISO-Sol). Afterwards, the metabolic activity was determined via WST-1 cell proliferation assay. The mean value of at least three independent experiments is shown. P-values at significance levels of ≤ 0.050 and ≤ 0.010 are indicated by (*) and (**), respectively. The cell lines GSU, H111TC and MKN7 were cetuximab sensitive, in contrast, HGC-27 displayed a cetuximab insensitive phenotype

Online Resource 2 Suspected copy number variations in GSU and H111TC cells

For H111TC cells, an *HER2* amplification is suspected by next generation sequencing analysis

Online Resource 3 Array-comparative genomic hybridization analysis for copy number status of locus *ERBB2* (*HER2*)

Array-comparative genomic hybridization analysis for copy number status of locus *ERBB2* (*HER2*) for cell lines GSU (a) and H111TC (b). Left panels: overview of the log2ratios of probes on chr17q12-chr17q21.32. Right panels: Magnification for locus *ERBB2*. Focal aberrations defined as size <3 Mb according to literature (Krijgsman et al., 2014). CEP copy number defined as the lower of the two mean copy numbers of the p- and q-arm. CEP17: centromere 17

Online Resource 4 Amplifications and deletions of selected genes in GSU and H111TC cells

Amplifications and deletions of selected genes in the gastric cancer cell lines GSU and H111TC determined by array-comparative genomic hybridization analysis

Online Resource 5 Effect of treatment with trastuzumab for 8 days on the expression profile of HER and pHER-receptors – densitometric measurement

All gastric cancer cell lines were treated for 8 days with 10 µg/ml trastuzumab, afterwards, the expression of EGFR, HER2, HER3, HER4, pEGFR, pHER2, pHER3, and pHER4 were determined via Western blot analysis. H111TC, HGC-27, MKN1 and MKN28 displayed a significant decrease in HER2 expression after trastuzumab treatment. pHER3 levels significantly decreased in Hs746T and MKN7 cells. The mean value of at least three independent experiments is shown. P-values at significance levels of ≤ 0.050 and ≤ 0.010 are indicated by (*) and (**), respectively

Online Resource 6 Effect of treatment with cetuximab for 8 days on the expression profile of HER and pHER-receptors – densitometric measurement

All gastric cancer cell lines were treated for 8 days with 10 µg/ml cetuximab, afterwards, the expression of EGFR, HER2, HER3, HER4, pEGFR, pHER2, pHER3, and pHER4 were determined via Western blot analysis. EGFR levels decreased significantly in MKN28 cells. Significant increases were detected for HER2 in H111TC cells and for HER2 and pHER2 in MKN7 cells. The mean value of at least three independent experiments is shown. P-values at significance levels of ≤ 0.050 and ≤ 0.010 are indicated by (*) and (**), respectively

Online Resource 7 Secretion of AREG in the gastric cancer cell lines GSU, H111TC, HGC-27 and MKN7

Cells were incubated for 24 h before the amount of secreted AREG was measured in the conditioned medium by ELISA-assay. GSU, H111TCand MKN7cells secreted high levels of AREG to the medium, AREG secretion was hardly detectable for HGC-27 cells. The mean value of at least three independent experiments is shown. P-values at significance levels of ≤ 0.050 and ≤ 0.010 are indicated by (*) and (**), respectively

Online Resource 9 Effect of exogenous ligand application on cetuximab sensitivity in MKN1 and Hs746T cells

MKN1 and Hs746T cells were treated for 3 days with100 µg/ml cetuximab and/or different HER receptor ligands (AREG: 7.5/15/150 ng/ml; EGF: 0.05/0.1/1 ng/ml; HB-EGF: 0.2/0.4/4 ng/ml). The metabolic activity of the cells was measured using the WST-1 cell proliferation assay. HB-EGF and EGF but not AREG were effective in rescuing MKN1 from cetuximab inhibition. The mean value of three independent experiments is shown. P-values at significance levels of ≤ 0.050 and ≤ 0.010 are indicated by (*) and (**), respectively

Reference

Krijgsman, O., Carvalho, B., Meijer, G.A., Steenbergen, R.D., and Ylstra, B. (2014). Focal chromosomal copy number aberrations in cancer-Needles in a genome haystack. Biochim Biophys Acta *1843*, 2698-2704.
