# Supplementary material for: Influence of the HER receptor ligand system on sensitivity to cetuximab and trastuzumab in gastric cancer cell lines
Source: J Cancer Res Clin Oncol. 2016 Dec 8;143(4):573–600. doi: 10.1007/s00432-016-2308-z (PMC5352771; doi:10.1007/s00432-016-2308-z)
Supplement: Supplementary file 2 — Supplementary material 2 (PDF 261 kb) [file 432_2016_2308_MOESM2_ESM.pdf]

Influence of the Her-receptor ligand system on sensitivity to cetuximab and trastuzumab in gastric cancer cell lines

Journal of Cancer Research and Clinical Oncology

Julia Kneissl, Anja Hartmann, Nicole Pfarr, Franziska Erlmeier, Thomas Lorber, Simone Keller, Gwen Zwingenberger, Wilko Weichert, Birgit Luber

Correspondence to: Dr. Birgit Luber, Institute of Pathology, Technische Universität München, Klinikum rechts der Isar, Trogerstr. 18, D-81675 München, Germany, E-mail: birgit.luber@tum.de

p-values

Legend
p ≤ 0,05
p ≤ 0,01

GSU
pEGFR

		1	2	3	4	5	6	7	8	9	10
		GSU_untreated	GSU_trastuzumab	GSU_AREG	GSU_trastuzumab/AREG	GSU_EGF	GSU_trastuzumab/EGF	GSU_HB-EGF	GSU_trastuzumab/HB-EGF	GSU_TGFα	GSU_trastuzumab/TGFα
1	GSU_untreated		0,038	0,816	0,011	0,611	0,021	0,317	0,42	0,021	0,11
2	GSU_trastuzumab	0,038		0,027	0,192	0,02	0,09	0,024	0,05	0,018	0,242
3	GSU_AREG	0,816	0,027		0,049	0,583	0,145	0,437	0,574	0,128	0,122
4	GSU_trastuzumab/AREG	0,011	0,192	0,049		0,015	0,163	0,005	0,159	0,002	0,955
5	GSU_EGF	0,611	0,02	0,583	0,015		0,044	0,812	0,312	0,176	0,074
6	GSU_trastuzumab/EGF	0,021	0,09	0,145	0,163	0,044		0,012	0,431	0,003	0,471
7	GSU_HB-EGF	0,317	0,024	0,437	0,005	0,812	0,012		0,244	0,128	0,071
8	GSU_trastuzumab/HB-EG	0,42	0,05	0,574	0,159	0,312	0,431	0,244		0,101	0,254
9	GSU_TGFa	0,021	0,018	0,128	0,002	0,176	0,003	0,128	0,101		0,043
10	GSU_trastuzumab/TGFa	0,11	0,242	0,122	0,955	0,074	0,471	0,071	0,254	0,043	

GSU
pHer2

		1	2	3	4	5	6	7	8	9	10
		GSU_untreated	GSU_trastuzumab	GSU_AREG	GSU_trastuzumab/AREG	GSU_EGF	GSU_trastuzumab/EGF	GSU_HB-EGF	GSU_trastuzumab/HB-EGF	GSU_TGFα	GSU_trastuzumab/TGFα
1	GSU_untreated		0,235	0,379	0,671	0,298	0,713	0,162	0,636	0,157	0,542
2	GSU_trastuzumab	0,235		0,145	0,296	0,134	0,287	0,102	0,168	0,101	0,419
3	GSU_AREG	0,379	0,145		0,035	0,518	0,057	0,085	0,614	0,059	0,21
4	GSU_trastuzumab/AREG	0,671	0,296	0,035		0,027	0,924	0,008	0,274	0,012	0,708
5	GSU_EGF	0,298	0,134	0,518	0,027		0,045	0,117	0,442	0,03	0,18
6	GSU_trastuzumab/EGF	0,713	0,287	0,057	0,924	0,045		0,015	0,304	0,02	0,68
7	GSU_HB-EGF	0,162	0,102	0,085	0,008	0,117	0,015		0,192	0,827	0,115
8	GSU_trastuzumab/HB-EG	0,636	0,168	0,614	0,274	0,442	0,304	0,192		0,181	0,314
9	GSU_TGFa	0,157	0,101	0,059	0,012	0,03	0,02	0,827	0,181		0,114
10	GSU_trastuzumab/TGFa	0,542	0,419	0,21	0,708	0,18	0,68	0,115	0,314	0,114	

MKN45
pEGFR

		1	2	3	4	5	6	7	8	9	10
		MKN45_untreated	MKN45_trastuzumab	MKN45_AREG	MKN45_trastuzumab/AREG	MKN45_EGF	MKN45_trastuzumab/EGF	MKN45_HB-EGF	MKN45_trastuzumab/HB-EGF	MKN45_TGFα	MKN45_trastuzumab/TGFα
1	MKN45_untreated		0,384	0,658	0,434	0,611	0,656	0,646	0,714	0,59	0,423
2	MKN45_trastuzumab	0,384		0,689	0,973	0,747	0,762	0,535	0,433	0,496	0,357
3	MKN45_AREG	0,658	0,689		0,719	0,946	0,969	0,579	0,542	0,534	0,383
4	MKN45_trastuzumab/ARE	0,434	0,973	0,719		0,77	0,774	0,533	0,434	0,494	0,355
5	MKN45_EGF	0,611	0,747	0,946	0,77		0,982	0,57	0,52	0,526	0,377
6	MKN45_trastuzumab/EGF	0,656	0,762	0,969	0,774	0,982		0,574	0,536	0,529	0,379
7	MKN45_HB-EGF	0,646	0,535	0,579	0,533	0,57	0,574		0,758	0,918	0,725
8	MKN45_trastuzumab/HB-	0,714	0,433	0,542	0,434	0,52	0,536	0,758		0,686	0,495
9	MKN45_TGFa	0,59	0,496	0,534	0,494	0,526	0,529	0,918	0,686		0,811
10	MKN45_trastuzumab/TGF	0,423	0,357	0,383	0,355	0,377	0,379	0,725	0,495	0,811	

MKN45
pHer2

		1	2	3	4	5	6	7	8	9	10
		MKN45_untreated	MKN45_trastuzumab	MKN45_AREG	MKN45_trastuzumab/AREG	MKN45_EGF	MKN45_trastuzumab/EGF	MKN45_HB-EGF	MKN45_trastuzumab/HB-EGF	MKN45_TGFα	MKN45_trastuzumab/TGFα
1	MKN45_untreated		0,664	0,791	0,973	0,966	0,839	0,477	0,816	0,674	0,845
2	MKN45_trastuzumab	0,664		0,649	0,467	0,638	0,734	0,521	0,583	0,289	0,332
3	MKN45_AREG	0,791	0,649		0,612	0,789	0,946	0,186	0,845	0,367	0,433
4	MKN45_trastuzumab/ARE	0,973	0,467	0,612		0,923	0,738	0,249	0,647	0,609	0,815
5	MKN45_EGF	0,966	0,638	0,789	0,923		0,853	0,418	0,821	0,597	0,774
6	MKN45_trastuzumab/EGF	0,839	0,734	0,946	0,738	0,853		0,429	0,992	0,443	0,577
7	MKN45_HB-EGF	0,477	0,521	0,186	0,249	0,418	0,429		0,168	0,187	0,173
8	MKN45_trastuzumab/HB-	0,816	0,583	0,845	0,647	0,821	0,992	0,168		0,384	0,46
9	MKN45_TGFa	0,674	0,289	0,367	0,609	0,597	0,443	0,187	0,384		0,746
10	MKN45_trastuzumab/TGF	0,845	0,332	0,433	0,815	0,774	0,577	0,173	0,46	0,746	

Figure legend

Online Resource 8 Effect of trastuzumab and concomitant ligand application on pEGFR and pHER2 levels in GSU and MKN45 cells – list of all significant p-values (referring to Fig. 8)

GSU and MKN45 cells were treated for 6 h with 10 µg/ml trastuzumab (Tra) and 15 ng/ml AREG (A) or 0.1 ng/ml EGF (E) or 0.4 ng/ml HB-EGF (H) or 0.75 ng/ml TGFα (T). Subsequently, pEGFR and pHER levels were determined by Western blot analysis.
